# Integrated holobiont responses of the Antarctic brown seaweed *Adenocystis utricularis* to thermal stress under a climate change scenario

**DOI:** 10.1093/ismeco/ycag208

**Published:** 2026-07-21

**Authors:** Kerina González-Pino, Agustina Undabarrena, Francisca Morales, Néstor Serna-Cardona, Pamela T Muñoz, Paula S M Celis-Plá, Daniela Rago, Verónica Molina, Nelso P Navarro, Claudio A Sáez, Céline Lavergne, Beatriz Cámara, Catherine Tessini, Gabriela B Pérez-Hernández, Jeniffer Pereira-Rojas, Linda Ahonen, Polette Aguilar-Muñoz, Fernanda Rodríguez-Rojas

**Affiliations:** HUB AMBIENTAL UPLA, Universidad de Playa Ancha, Valparaíso 2360004, Chile; Doctorado Interdisciplinario en Ciencias Ambientales, Facultad de Ciencias Naturales y Exactas, Universidad de Playa Ancha, 2360004 Valparaíso, Chile; Laboratorio Observatorio de Ecología Microbiana LABOEM, HUB AMBIENTAL UPLA, Universidad de Playa Ancha, Valparaíso 2360004, Chile; Novo Nordisk Foundation Center for Biosustainability, Technical University of Denmark, Kongens Lyngby 2800, Denmark; ActinoLab USM, Centro de Biotecnología “Daniel Alkalay Lowitt”, Valparaíso 2390123, Chile; HUB AMBIENTAL UPLA, Universidad de Playa Ancha, Valparaíso 2360004, Chile; ActinoLab USM, Centro de Biotecnología “Daniel Alkalay Lowitt”, Valparaíso 2390123, Chile; Departamento de Química, Universidad Técnica Federico Santa María, Valparaíso 2390123, Chile; Departamento de Ciencias Biológicas, Instituto de Ciencias Biomédicas, Facultad de Ciencias de la Salud, Universidad Autónoma de Chile, Talca 3460000, Chile; HUB AMBIENTAL UPLA, Universidad de Playa Ancha, Valparaíso 2360004, Chile; Laboratorio de Investigación Ambiental Acuático, HUB AMBIENTAL UPLA, Universidad de Playa Ancha, Valparaíso 2360004, Chile; Departamento de Ciencia y Geografía, Facultad de Ciencias Naturales y Exactas, Universidad de Playa Ancha, Valparaíso 2360004, Chile; Novo Nordisk Foundation Center for Biosustainability, Technical University of Denmark, Kongens Lyngby 2800, Denmark; HUB AMBIENTAL UPLA, Universidad de Playa Ancha, Valparaíso 2360004, Chile; Laboratorio Observatorio de Ecología Microbiana LABOEM, HUB AMBIENTAL UPLA, Universidad de Playa Ancha, Valparaíso 2360004, Chile; Departamento de Ciencia y Geografía, Facultad de Ciencias Naturales y Exactas, Universidad de Playa Ancha, Valparaíso 2360004, Chile; Centro de Investigación Oceanográfica COPAS COASTAL, Universidad de Concepción, Concepción 3349001, Chile; Laboratorio de Ecofisiología y Biotecnología de Algas (LEBA), Facultad de Ciencias, Universidad de Magallanes, 6200000 Punta Arenas, Chile; Network for Extreme Environments Research, NEXER-Universidad de Magallanes, 6200000 Punta Arenas, Chile; Multidisciplinary Institute for Environmental Studies (IMEM) Ramón Margalef, University of Alicante, 03080 Alicante, Spain; Department of Marine Sciences and Applied Biology, University of Alicante, 03690 Alicante, Spain; Laboratorio de Investigación Ambiental Acuático, HUB AMBIENTAL UPLA, Universidad de Playa Ancha, Valparaíso 2360004, Chile; Departamento de Ciencia y Geografía, Facultad de Ciencias Naturales y Exactas, Universidad de Playa Ancha, Valparaíso 2360004, Chile; Departament de Biologia Marina i Oceanografia, Institut de Ciències del Mar, ICM-CSIC, Barcelona 08003, Spain; Laboratorio Observatorio de Ecología Microbiana LABOEM, HUB AMBIENTAL UPLA, Universidad de Playa Ancha, Valparaíso 2360004, Chile; Laboratorio de Investigación Ambiental Acuático, HUB AMBIENTAL UPLA, Universidad de Playa Ancha, Valparaíso 2360004, Chile; Departamento de Ciencia y Geografía, Facultad de Ciencias Naturales y Exactas, Universidad de Playa Ancha, Valparaíso 2360004, Chile; ActinoLab USM, Centro de Biotecnología “Daniel Alkalay Lowitt”, Valparaíso 2390123, Chile; Departamento de Química, Universidad Técnica Federico Santa María, Valparaíso 2390123, Chile; Laboratorio de Electroquímica y Química Analítica, Departamento de Química, Universidad Técnica Federico Santa María, Valparaíso 2390123, Chile; HUB AMBIENTAL UPLA, Universidad de Playa Ancha, Valparaíso 2360004, Chile; Doctorado Interdisciplinario en Ciencias Ambientales, Facultad de Ciencias Naturales y Exactas, Universidad de Playa Ancha, 2360004 Valparaíso, Chile; HUB AMBIENTAL UPLA, Universidad de Playa Ancha, Valparaíso 2360004, Chile; Doctorado Interdisciplinario en Ciencias Ambientales, Facultad de Ciencias Naturales y Exactas, Universidad de Playa Ancha, 2360004 Valparaíso, Chile; Novo Nordisk Foundation Center for Biosustainability, Technical University of Denmark, Kongens Lyngby 2800, Denmark; HUB AMBIENTAL UPLA, Universidad de Playa Ancha, Valparaíso 2360004, Chile; Laboratorio Observatorio de Ecología Microbiana LABOEM, HUB AMBIENTAL UPLA, Universidad de Playa Ancha, Valparaíso 2360004, Chile; Departamento de Ciencia y Geografía, Facultad de Ciencias Naturales y Exactas, Universidad de Playa Ancha, Valparaíso 2360004, Chile; Centro de Investigación Oceanográfica COPAS COASTAL, Universidad de Concepción, Concepción 3349001, Chile; HUB AMBIENTAL UPLA, Universidad de Playa Ancha, Valparaíso 2360004, Chile; Laboratorio Observatorio de Ecología Microbiana LABOEM, HUB AMBIENTAL UPLA, Universidad de Playa Ancha, Valparaíso 2360004, Chile; Laboratorio de Investigación Ambiental Acuático, HUB AMBIENTAL UPLA, Universidad de Playa Ancha, Valparaíso 2360004, Chile; Departamento de Ciencia y Geografía, Facultad de Ciencias Naturales y Exactas, Universidad de Playa Ancha, Valparaíso 2360004, Chile

**Keywords:** macroalgae, holobiont, microbiome, metabolome, biomarkers, climate change, Antarctica

## Abstract

The Antarctic intertidal zone hosts ecologically pivotal holobionts such as the brown macroalga *Adenocystis utricularis*, whose response to climate-driven stressors remains poorly understood. Here, we applied an integrative holobiont framework combining microbial community profiling, host physiological assays, and phycosphere metabolomics to assess how thermal stress (2°C vs. 8°C) influences microbiome dynamics, host stress responses, and algal surface chemistry after 5 days under experimental conditions. Results showed that warming induced a transient reduction in microbial diversity, with Shannon diversity decreasing at Day 3 under 8°C and returning to levels comparable to the control by Day 5, accompanied by marked community structural reorganization. Elevated temperature enriched Campylobacteria and Gammaproteobacteria, while Bacteroidia and Verrucomicrobia decreased. Functional predictions revealed a shift from nutrient cycling and carbon turnover at 2°C toward heterotrophy, fermentation, and bacterivory-related pathways at 8°C. Microbiome disruption was associated with impaired photosynthetic performance, increased oxidative damage, reduced antioxidant defenses, and altered osmolyte accumulation, under warming conditions. Untargeted metabolomics uncovered pronounced thermal reprogramming of the algal surface metabolome, with >98% of detected features remaining chemically uncharacterized. Detected key metabolites included ceramides, N-acyl amino acids, and amphipathic compounds with putative cytotoxic or antioxidant activities, many of which increased under 8°C. Together, these findings demonstrate that *A. utricularis* response to thermal stress emerges from dynamic host–microbiome–metabolome interactions. By linking microbial restructuring, host physiology, and metabolomic plasticity, our study highlights the holobiont as the operative unit of adaptation in Antarctic coastal ecosystems facing climate change.

## Graphical Abstract

**Figure:**
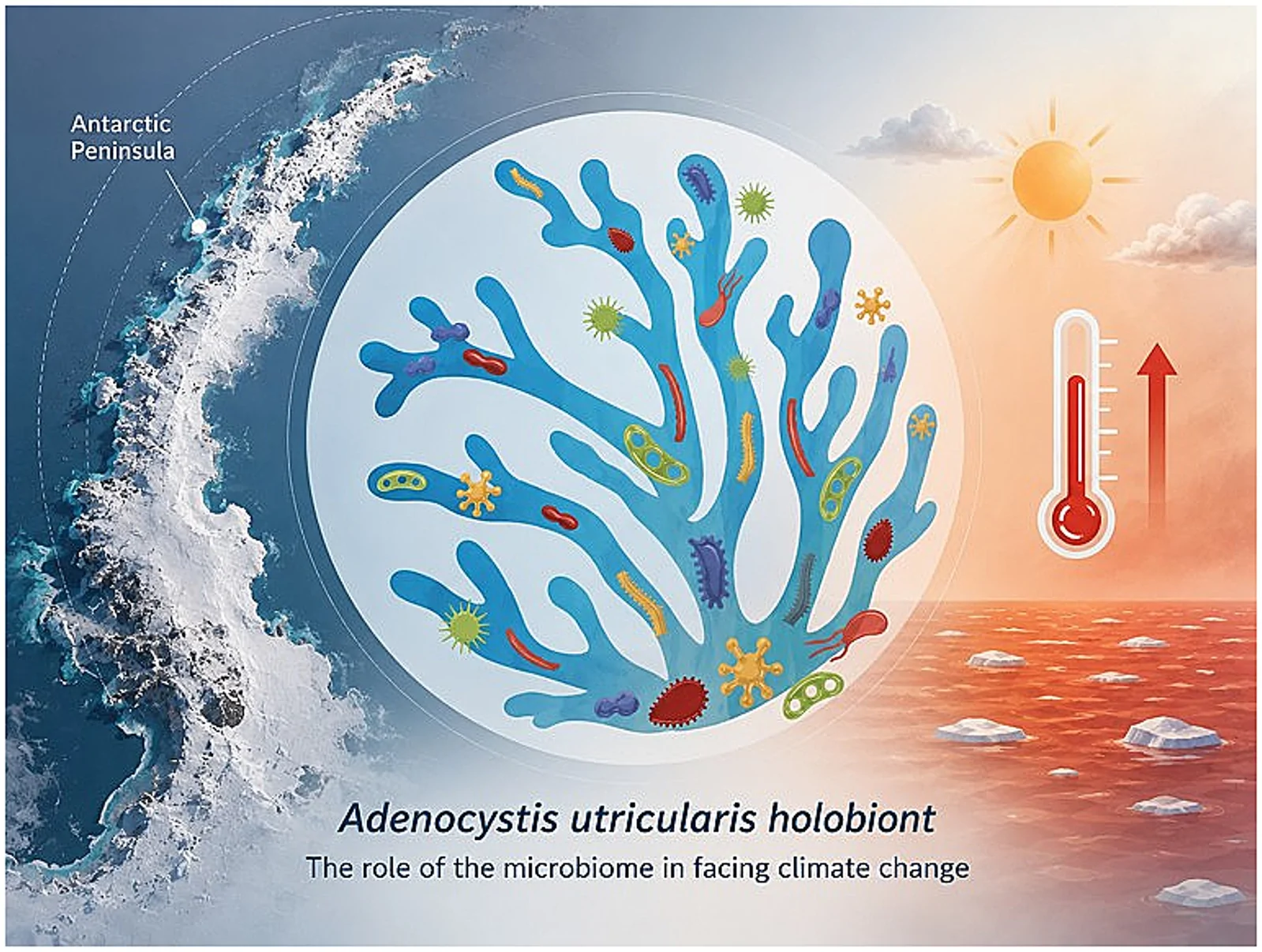

## Introduction

The Antarctic intertidal zone represents a dynamic interface between land and sea, supporting ecologically significant holobiont primary producers such as macroalgae. These holobionts play foundational roles in polar coastal ecosystems by contributing to primary productivity, nutrient cycling, and carbon sequestration [1, 2]. However, this ecosystem is increasingly susceptible to climate-related stressors such as, salinity, increased UV exposure, and rising temperatures [3, 4]. Currently, the sea surface temperature in this region is ~2°C, but regional climate change projections for nearshore and intertidal zone indicate that, under high-emission scenarios, temperature may reach values approaching 6°C–8°C by 2100 [4]. In addition to long-term ocean warming, polar regions are increasingly experiencing marine heatwaves, characterized by short-term thermal anomalies lasting from several days to up to a month and reaching temperature increases of ~+1 to +3°C above the local mean sea surface temperature [5]. These episodic events are becoming more frequent and intense in high-latitude oceans, with growing evidence that they can impose acute physiological stress on cold-adapted marine organisms [6].

Recent research has underscored the remarkable physiological plasticity of Antarctic macroalgae to elevated temperatures [7]. However, experimental evidence suggests that such thermal tolerance may not be an intrinsic property of the host alone, but rather an emergent feature of the macroalgal holobiont [8]. In particular, host-associated microbial communities have been shown to modulate metabolic performance and stress resilience under projected warming scenarios [8], raising the question of whether microbiome integrity is a key determinant of thermal tolerance in Antarctic macroalgae. Beyond physiological adaptations of the seaweed host, increasing attention has been directed to the role of the macroalgal microbiome in shaping host tolerance and resilience [9]. The holobiont framework emphasizes that macroalgae and their associated microbial communities form integrated ecological units, with microbial partners playing critical roles in host defense, metabolism, and environmental tolerance [8–10]. For example, the red seaweed *Gracilaria vermiculophylla* can selectively recruit beneficial epiphytic bacteria that produce antimicrobial metabolites, inhibiting pathogens like *Kordia algicida* [11]. Macroalgal surfaces support dense and diverse microbial biofilms that modulate both abiotic interactions and host physiological processes [8, 12]. However, environmental stressors can disrupt this selection process, leading to dysbiosis and compromising holobiont health [13]. For instance, changes in salinity and temperature have been shown to destabilize microbial communities associated with *G. vermiculophylla*, correlating with impaired host performance [11, 13]. Under such conditions, the stochastic assembly of microbiota can increase, in line with the “Anna Karenina principle”, which posits that stressed hosts exhibit greater microbiome variability due to a breakdown in selective control of microorganisms [14–16]. Despite their ecological relevance, studies on epiphytic microbiota in Antarctic macroalgae remain scarce, with current knowledge largely limited to culturable Gram-positive bacteria [17]. Even less is known about the chemical dimension of these interactions, particularly the surface metabolome. Surface-associated metabolites may mediate microbial colonization, act as chemical defenses, or modulate tolerance to environmental stressors, and are therefore likely fundamental to holobiont stability [18]. However, their chemical diversity, ecological roles, and interactions with epiphytic microbiota remain largely unexplored in polar systems.

Within this context, the Antarctic brown macroalga *Adenocystis utricularis* represents an ecologically relevant and well-established model to investigate holobiont responses to environmental change. This species is widely distributed across Antarctic and sub-Antarctic coastal systems, where it functions as a habitat-forming primary producer in intertidal and shallow subtidal zones. Recent experimental studies have demonstrated its capacity to cope with combined stressors such as temperature and salinity, revealing pronounced physiological flexibility and adaptive stress-response mechanisms under climate-relevant conditions [7, 19]. Together, its broad geographic distribution, ecological importance, and documented stress tolerance make *A. utricularis* a suitable model to explore how microbiome dynamics and chemical interactions contribute to holobiont resilience in rapidly changing polar environments.

In this study, we used the Antarctic brown macroalga *A. utricularis* as a holobiont model to investigate the dynamics of epiphytic microbial communities and their functional role in thermal stress tolerance, under a controlled laboratory experiment. First, we characterized the composition and structure of the microbiota under different temperature regimes using 16S rRNA gene metabarcoding to assess shifts in the active community in response to warming. Then, we selectively disrupt the microbial communities using an antimicrobial cocktail to assess their contribution to host health and tolerance (i.e. photosynthetic performance and stress biomarkers). Finally, we analyzed the algal surface metabolome using untargeted metabolomic approaches to identify chemical signatures potentially involved in microbial interactions and thermal resilience. This integrative approach allowed us to link microbiome dynamics and metabolomic shifts to the physiological responses of the host under climate change–projected ocean warming scenarios.

## Materials and methods

### Samples collection and culture conditions

Specimens of *A. utricularis* were gathered in plastic containers from the intertidal zone of Punta Artigas, located in Bahía Fildes, King George Island, Antarctica (62°11′06.2′′S 58°52′42.9′′W) during the austral summer of 2023 (Supplementary Fig. 1). After collection, the algal fronds were rinsed twice using filtered seawater (0.22 μm) and kept in plastic boxes for 48 h at 2°C in complete darkness with continuous aeration to allow acclimation to laboratory conditions. Following this period, ~300 mg of fresh biomass was transferred into 1 L glass bottles filled with filtered seawater (0.22 μm), considering one bottle as a biological replicate (six bottles per water bath). Each experimental bottle was considered an independent replicate and contained multiple individual algal thalli; at each sampling point, tissue from several individuals within the same bottle was pooled for further analyses. The experimental setup involved thermoregulated baths maintained at 2°C (control) and 8°C (thermal stress), following the methodology described by [7, 19]. All bath temperatures were monitored using a Checktemp Digital Thermometer HI98509-1 (Hanna Instruments). Three bottles within each bath were submitted daily to the addition of an antimicrobial cocktail to disrupt the microbial load of the holobiont samples. Antimicrobial cocktail consisted in: neomycin (500 μg ml^−1^), ampicillin (250 μg ml^−1^), kanamycin (100 μg ml^−1^), cycloheximide (100 μg ml^−1^), streptomycin (50 μg ml^−1^) and germanium dioxide (1 μg ml^−1^). Antimicrobial composition and dosing were selected based on previous studies in marine macroalgae and seagrasses, using concentrations shown to disrupt associated microbiota while minimizing direct physiological effects on the algal host [20–22]. The samples were subjected to a 20:4 light–dark photoperiod, with photosynthetically active radiation of 90 μmol m^2^ s^−1^ and constant filtered air (0.22 μm) aeration. Sampling for photosynthetic parameters (*F_v_/F_m_*, ETR and NPQ), 16S rRNA metabarcoding and biomarkers (ROS, protein carbonylation, lipid peroxidation, ascorbate, free amino acids, and proline) were collected at 0 (d0), 3 (d3), and 5 (d5) days. For metabolomic analyses samples were collected only at d5 (both temperatures) and compared to native (*in situ*) samples collected at Punta Artigas, due to the large amount of biomass required for this type of analyses. Epiphytic microbiomes were sampled by rubbing 5 cm^2^ of the algae frond using sterilized cotton swabs under a biosafety cabinet, preserved in 300 μL of RNALater and stored at −20°C. For biomarkers and metabolome analyses samples were rinsed twice with filtered distilled water, rapidly frozen in liquid nitrogen, and preserved at −80°C. After finished the experiment all samples were transported in a dry shipper container from Antarctica to the HUB AMBIENTAL UPLA in Valparaíso, Chile. Upon arrival, they were stored at −80°C until further analysis.

### Microbial community analyses

The active epiphytic microbial communities were studied using RNA-based approaches. Total RNA was extracted from epiphytic samples using the mirVana™ miRNA Isolation Kit (Thermo Fisher Scientific), with modifications adapted from the protocol described in [23]. Residual genomic DNA was removed using TURBO DNase (Thermo Fisher Scientific) following the manufacturer’s instructions. Complementary DNA was subsequently synthesized using the ImProm-II™ Reverse Transcription System (Promega) according to the manufacturer’s protocol. High-throughput sequencing of the 16S rRNA V4 region using primers 515F/806R [24] was performed on the Illumina NovaSeq PE250 platform (Novogene).

Sequence curation and analyses were conducted using QIIME2 v.2021.11 [25]. Low-quality and short reads were removed resulting in an average of 158 081 ± 43 960 sequences per sample (224–403 bp; ~92.1 ± 0.8% of total reads). Amplicon sequence variants (ASVs) were generated using the DADA2 algorithm implemented in QIIME2 with the “denoise-paired” function, which performs error correction, dereplication, chimera removal, and inference of exact ASVs [26]. Reads assigned to chloroplast, mitochondria, and unassigned taxa were removed prior downstream analyses. ASVs were generated, and taxonomic assignment was performed using the SILVA 138 reference database with “vsearch” algorithm [27]. Raw sequence data are available in the European Nucleotide Archive under the BioProject reference PRJEB109277.

Microbial community analyses, including alpha diversity indices (phyloseq [28]), differential ASV analysis (DESeq2 [29]), and functional predictions (microeco [30]) based on the reference FAPROTAX database [31], were conducted in RStudio v4.2.2. To account for differences in sequencing depth, samples were rarefied to an even depth prior to diversity and community structure analyses. Microbial community composition was explored using ordination-based approaches and relative abundance visualizations. Further details on microbial community composition visualization and statistical analyses are provided in the Supplementary Methods section (Supplementary Tables 1-10). Statistical analyses included one-way ANOVA for univariate data and PERMANOVA for multivariate community structure based on Bray–Curtis dissimilarities [32, 33]. PERMANOVA was conducted using one-factor models, testing the effects of temperature or antibiotic treatment independently. Sampling time was not included as an interaction term and was evaluated separately to avoid overparameterization.

### Photosynthetic performance and stress biomarkers

#### Photosynthetic parameters

Maximum Quantum Yield of PSII (*F_v_/F_m_*), Electron Transport Rate (ETR), and Non-photochemical quenching (NPQ), were determined as described in [7]. Further details are provided in the Supplementary Methods section.

#### Stress biomarkers

Total ROS, lipid peroxidation, protein carbonylation, ascorbate, free amino acid, and proline, were determined as described in [7, 34]. All statistical analyses were performed as described in [7]. Further details are provided in the Supplementary Methods section.

### Surface metabolome of *A. utricularis* analyses

Optimization of metabolome extraction analyses using solvent-dipping protocol are described in Supplementary methods and Supplementary Fig. 2. An integrative chemoinformatic approach was employed to investigate the algae surface metabolome of both experimental temperatures (2°C and 8°C) and control (native), considering three and four independent biological individuals, respectively, with four technical replicates each (*n* = 40). Samples were analysed by ultra-high performance liquid chromatography tandem mass spectrometry (UHPLC-MS/MS) using a Vanquish Duo UHPLC system (ThermoFisher Scientific) coupled to a Orbitrap IDX™ mass spectrometer (ThermoFisher Scientific). Further details of the procedure are provided in the Supplementary Methods section. Data preprocessing was performed using MZmine v2.53 [35] and output was explored with MetaboAnalyst [36] to construct a Principal Component Analysis plot and a Variable Importance Projection (VIP) analysis. Metabolic profiles were compared by their most relevant features intensities (peak area values) by hierarchical clustering heatmap and independent bar plots. All data visualizations were constructed by curating the outputs and creating plots using Python in combination with Matplotlib, Seaborn, Pandas, and NumPy packages. Samples MS/MS were submitted to the Global Natural Products Social Molecular Networking [37] using the GNPS-2 environment (https://gnps2.org/homepage) for Feature-Based Molecular Networking (FBMN) workflow [38], visualized and further edited with Cytoscape v3.9.1 [39]. For dereplication, MS/MS spectra were analysed with SIRIUS v4 [40], integrating CSI:Finger ID and CANOPUS databases. Additional manual searches were conducted in MassBlank database.

### Chemical–taxonomic contextualization using MicrobeMASST

For chemical–taxonomic contextualization, MS/MS spectra corresponding to selected metabolomic features were individually queried against public metabolomics datasets available in GNPS/MassIVE using MicrobeMASST [41]. Briefly, MicrobeMASST compares fragmentation spectra against publicly available MS/MS datasets and retrieves datasets containing similar spectral signatures together with their associated metadata and reported microbial taxa. Thus, the approach does not directly correlate ASV abundances with metabolite intensities from our experimental samples, but instead identifies taxonomic contexts in which similar metabolites have previously been detected across independent datasets.

Only spectral matches with cosine similarity ≥0.85 and at least six matched fragment ions were retained. Hits originating from blanks or quality-control samples were excluded. Taxonomic annotations associated with matching datasets were manually curated, standardized to the genus level when possible, and grouped into broad biological categories. Molecular features were subsequently grouped according to their FBMN-derived molecular networks, and a Network × Genus co-occurrence matrix was constructed based on the recurrence of spectral matches across independent datasets. The resulting matrix was visualized as a heatmap to highlight recurrent chemical–taxonomic associations.

## Results

### *Adenocystis utricularis* epiphytic microbiome structure dynamics under thermal stress

**Figure 1:**
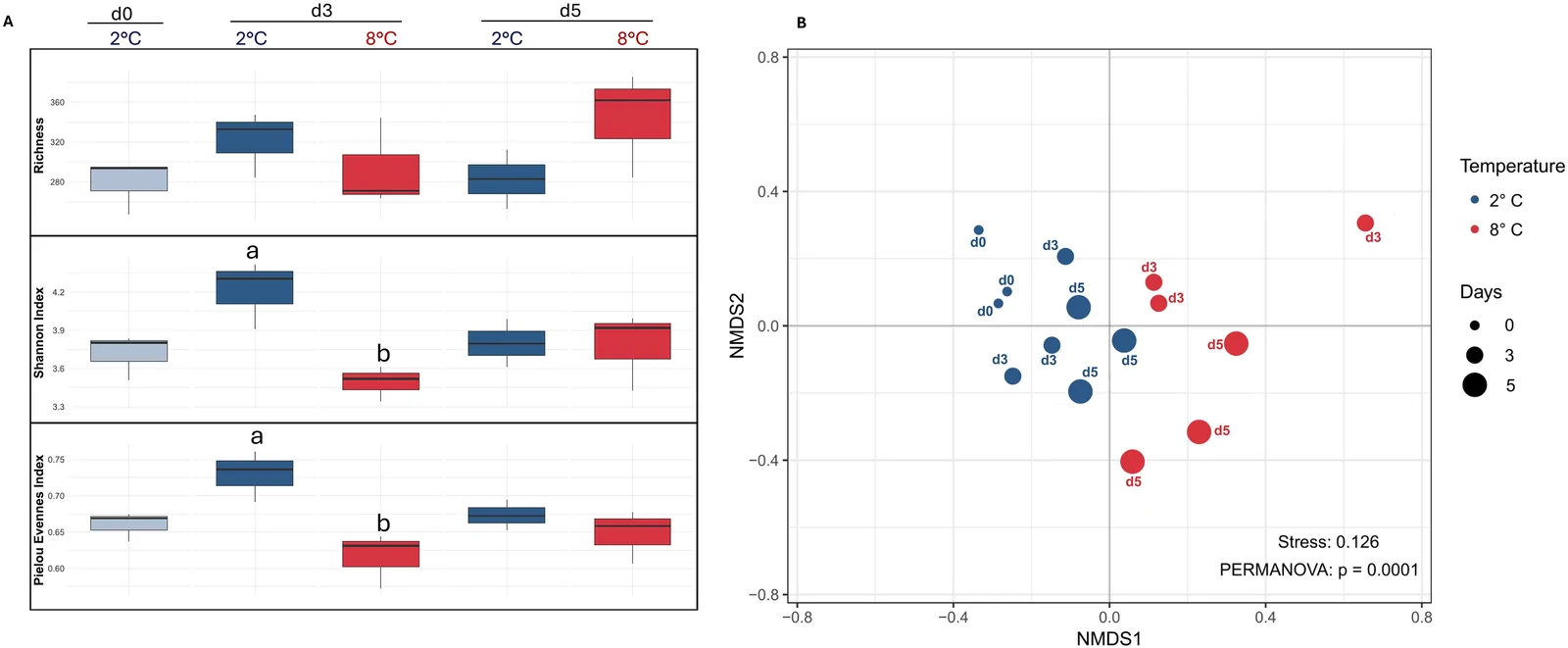
Epiphytic bacterial microbiome alpha and beta diversity in *a utricularis* under thermal exposure. (A) Alpha diversity indexes richness, Shannon and Pielou at day 0 post-acclimation, 2°C, and 8°C. Different letters indicate statistically significant differences between samples (HSD test, *P* < .05). (B) Bray–Curtis based NMDS analysis (stress = 0.126, PERMANOVA *P* < .05). Symbol size increases with treatment duration, with smaller symbols representing Day 3 and larger symbols representing Day 5. In all analyses a *n* = 3 was used.

Active microbial community alpha-diversity shifts were observed among treatments compared with initial conditions, especially for Shannon diversity indexes at Day 3 (Fig. 1A). Bacterial richness did not differ significantly between temperature treatments on Day 3, but significant differences were detected on Day 5. In contrast, the Shannon index exhibited a significant decline at 8°C on Day 3, consistent with an early alteration in community diversity patterns, accompanied by differences in taxonomic composition and predicted functional profiles. By Day 5 at 8°C, Shannon diversity index returned to levels comparable to the control, although differences in richness persisted among treatments. This rapid recovery in Shannon diversity suggests the persistence or rapid re-establishment of a resilient fraction of the epiphytic microbiome, although a full return to the initial community state (i.e. the original taxonomic composition and relative abundance structure observed prior to stress exposure) was not accomplished. A similar trend was observed for Pielou’s evenness index, which decreased under thermal stress on Day 3, but recovered partially by Day 5 (Fig. 1A). Microbial community structure (Fig. 1B) differed significantly among experimental conditions (PERMANOVA, *P* < .05), with ordination patterns showing clear separation between incubated samples at 2°C and 8°C.

**Figure 2:**
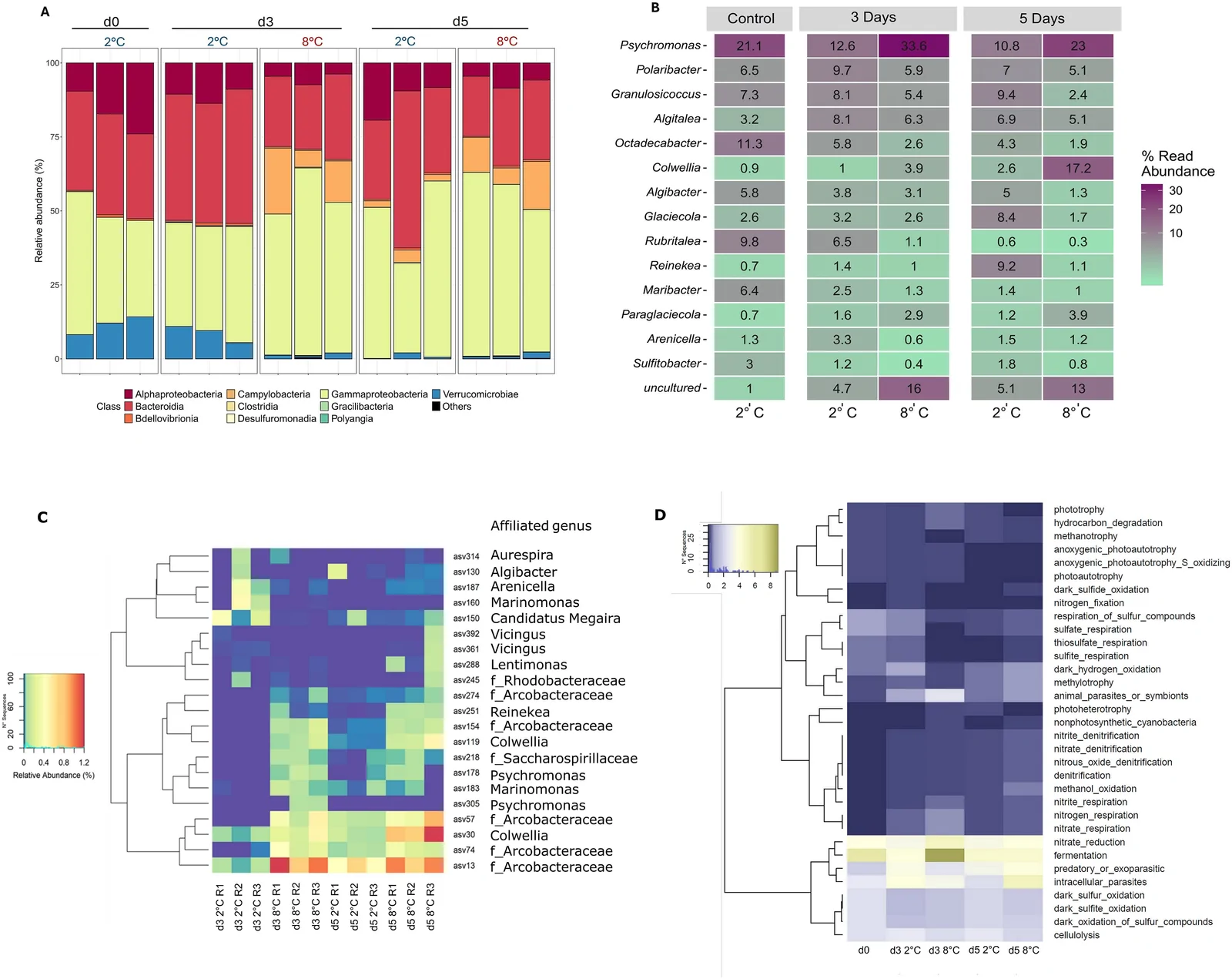
Epiphytic bacterial microbiome composition, structure in *A. utricularis* under thermal exposure. (A) Relative abundance of bacteria communities in *A. utricularis* epiphytic samples showing the 10 most abundant classes at 2°C and 8°C after 3 and 5 days of treatment. (B) Displays the 14 most abundant genera under the same conditions. (C) Differentially abundant ASVs (α = 0.01) across temperature treatments, with temporal trends indicated for each condition. T1 corresponds to samples collected after 3 days, and T2 to those collected after 5 days. (D) Taxonomy-based functional profiles of bacterial communities associated with *A. utricularis*. d0 corresponds to day 0 post-acclimation, d3 to samples collected after 3 days, and d5 to those collected after 5 days.

The taxonomic profiling of the active epiphytic microbial communities associated with *A. utricularis*, revealed that the bacterial classes Alphaproteobacteria, Bacteroidia, Gammaproteobacteria, and Verrucomicrobiae were consistently dominant across experimental conditions and time points (Fig. 2A). The persistence of these dominant classes across time points, together with the recurrent detection of genera such as *Granulosicoccus*, *Octadecabacter*, *Algibacter*, and *Colwellia*, is consistent with the presence of a subset of the epiphytic community that remains stable or rapidly reassembles under short-term thermal stress. However, thermal exposure at 8°C was associated with changes in the active microbial community composition. Specifically, a significant increase in the contribution of Campylobacteria and Gammaproteobacteria activity was detected at 8°C, accompanied by a marked decrease in the relative abundance of Bacteroidia and Verrucomicrobiota when compared to the control at both timepoints (Fig. 2A). Temperature-driven alterations were also evident among the fourteen most highly active bacterial taxa at the genus level (Fig. 2B). On Day 3, samples incubated at 2°C were dominated by the activity of *Granulosicoccus*, *Rubritalea*, *Polaribacter*, *Octadecabacter*, *Maribacter*, *Arenicella*, and *Sulfitobacter*. In contrast, the 8°C treatment showed higher relative activity of *Psychromonas*, *Colwellia*, and *Paraglaciecola*. By Day 5, the dominant genera at 2°C shifted to *Granulosicoccus*, *Octadecabacter*, *Algibacter*, *Glaciecola*, and *Reinekea*, whereas the microbial composition at 8°C remained relatively stable and similar to that observed on Day 3. Notably, a general increase in the relative abundance of *Colwellia* and *Paraglaciecola* was observed under sustained elevated temperature.

Differential abundance analyses (α = 0.01) provided further evidence of temperature-associated shifts in active microbial community composition (Fig. 2C). On Day 3, ASVs affiliated with Arcobacteraceae family, *Reinekea*, *Colwellia*, Saccharospirillaceae family, *Psychromonas*, and *Marinomonas*, were significantly retrieved at 8°C, whereas ASVs associated with *Arenicella* and *Candidatus Megaira* were more abundant at 2°C. This compositional divergence persisted through Day 5, although a general but comparatively lower enrichment of these ASVs was also detected at 2°C. Notably, ASVs affiliated to *Arenicella*, *Aurespira*, *Algibacter*, and *Marinomonas* (specifically ASV160) were found to be showed marked reductions in activity under 8°C conditions.

Taxonomy-based functional predictions (Fig. 2D) revealed distinct shifts in the potential metabolic functions of the epiphytic microbiome associated with *A. utricularis* in response to temperature. After 3 days of exposure, predicted functional traits such as nitrogen fixation, sulphur oxidation and respiration, cellulolysis, methylotrophy, and methanotrophy were increased at 2°C. In contrast, communities exposed to 8°C displayed an increased abundance of bacterial genera associated with fermentation, nitrate reduction, photoheterotrophy, hydrocarbon degradation, and parasites. By Day 5, the 2°C condition maintained high functional potential for cellulolysis and sulphur oxidation, while the 8°C treatment exhibited increased signals for predation, nitrogen metabolism, sulphur respiration, methylotrophy, and hydrogen oxidation.

### Health status in *A. utricularis* under thermal stress and microbiome disruption

**Figure 3:**
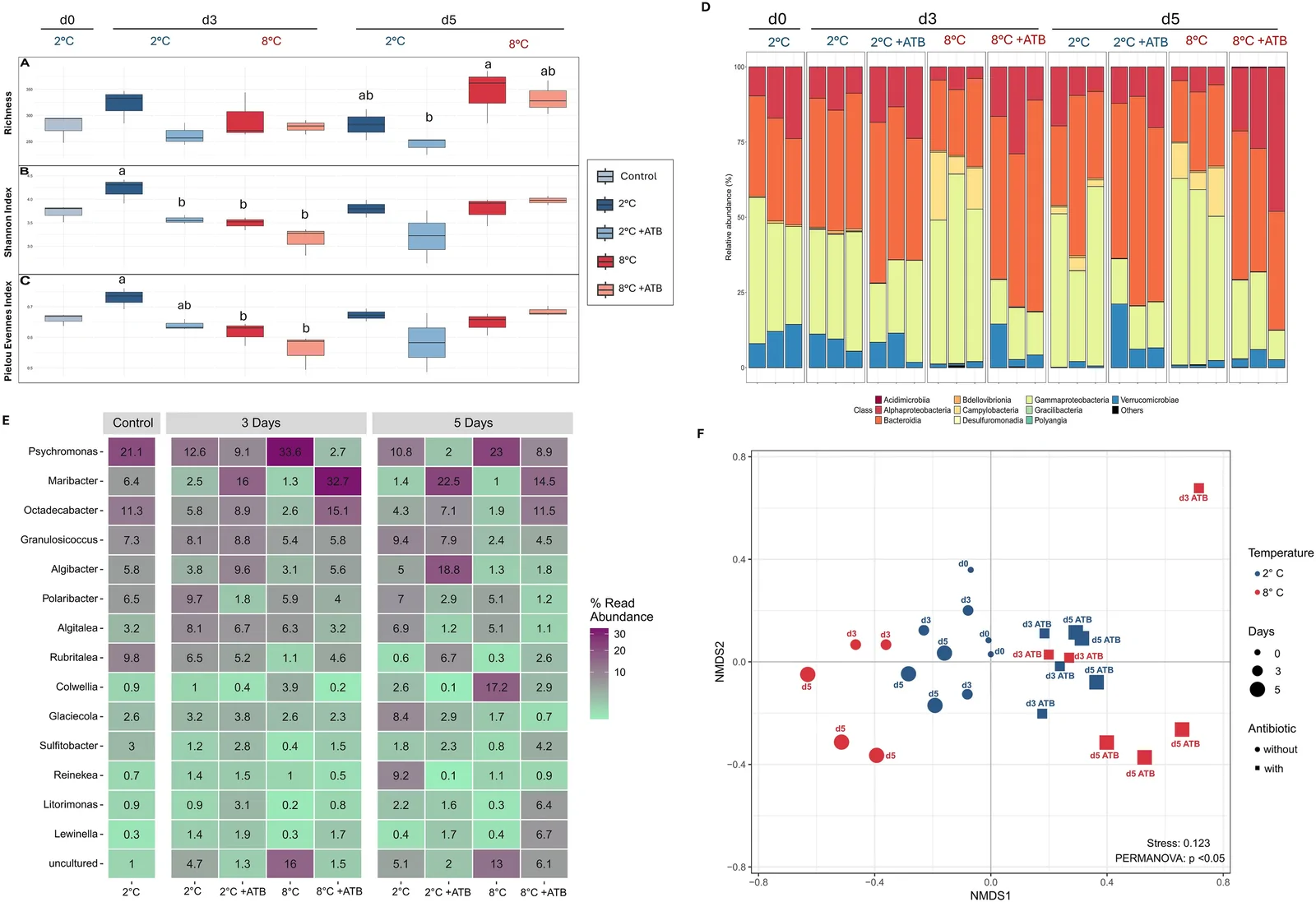
Epiphytic bacterial microbiome disruption under the exposure to antimicrobial cocktail and thermal stress in *A. utricularis*. Alpha diversity indexes: (A) Richness; (B) Shannon, and (C) Pielou. (D) Relative abundance of bacterial classes. Different letters indicate statistically significant differences between samples (HSD test, *P* < .05). (E) Differentially abundant ASVs (α = 0.01) across temperature treatments, with temporal trends indicated for each condition. (F) Bray–Curtis based NMDS analysis (stress = 0.126, PERMANOVA *P* < .05). Square symbols represent +ATB treatments. d0 corresponds to day 0 post-acclimation, d3 to samples collected after 3 days, and d5 to those collected after 5 days.

Health status in *A. utricularis* under thermal stress was measured through photosynthetic parameters and cellular stress biomarkers with (+ATB) and without (–ATB) microbiome disruption. Exposure to the antimicrobial cocktail was associated with a marked disruption of the active microbial assemblages, characterized by significant changes in alpha diversity and overall community composition, together with overall active community structure (Fig. 3). Alpha diversity was significantly decreased in samples treated with ATB on Day 3, especially at 2°C (Fig. 3A–C). In relative abundance, more pronounced shifts in community composition were observed, where ATB increased Bacteroidia class and diminished Campylobacteria in both temperatures and days (Fig. 3D).

Active taxa such as *Psychromonas*, *Colwellia*, *Algitalea*, and *Polaribacter*, which exhibited high activity at 8°C, were markedly suppressed under +ATB conditions (Fig. 3E). In contrast, *Maribacter*, *Octadecabacter*, *Granulosicoccus*, *Algibacter*, *Sulfitobacter*, *Litorimonas*, and *Lewinella* increased in relative abundance following antibiotic treatment. After 5 days, these patterns largely persisted. Notably, *Glaciecola* declined sharply at both temperatures, while *Litorimonas* decreased at 2°C but increased at 8°C (Fig. 3E). On the other hand, the microbial community structure of the antibiotic-treated group showed a significant distinct clustering pattern, with samples clearly separated from those without antibiotics (Fig. 3F). Notably, temperature-related differences were not detected as a significant structuring factor within this group. Taxonomy-based functional profiles predictions indicated that most affected predicted functions upon +ATB were associated with nitrogen, sulfur cycle and bacterivory (i.e. nitrate reduction, nitrate/nitrite denitrification, nitrogen respiration, nitrous oxide denitrification, sulfur compounds respiration and predatory, intracellular parasite) (Supplementary Fig. 3).

**Figure 4:**
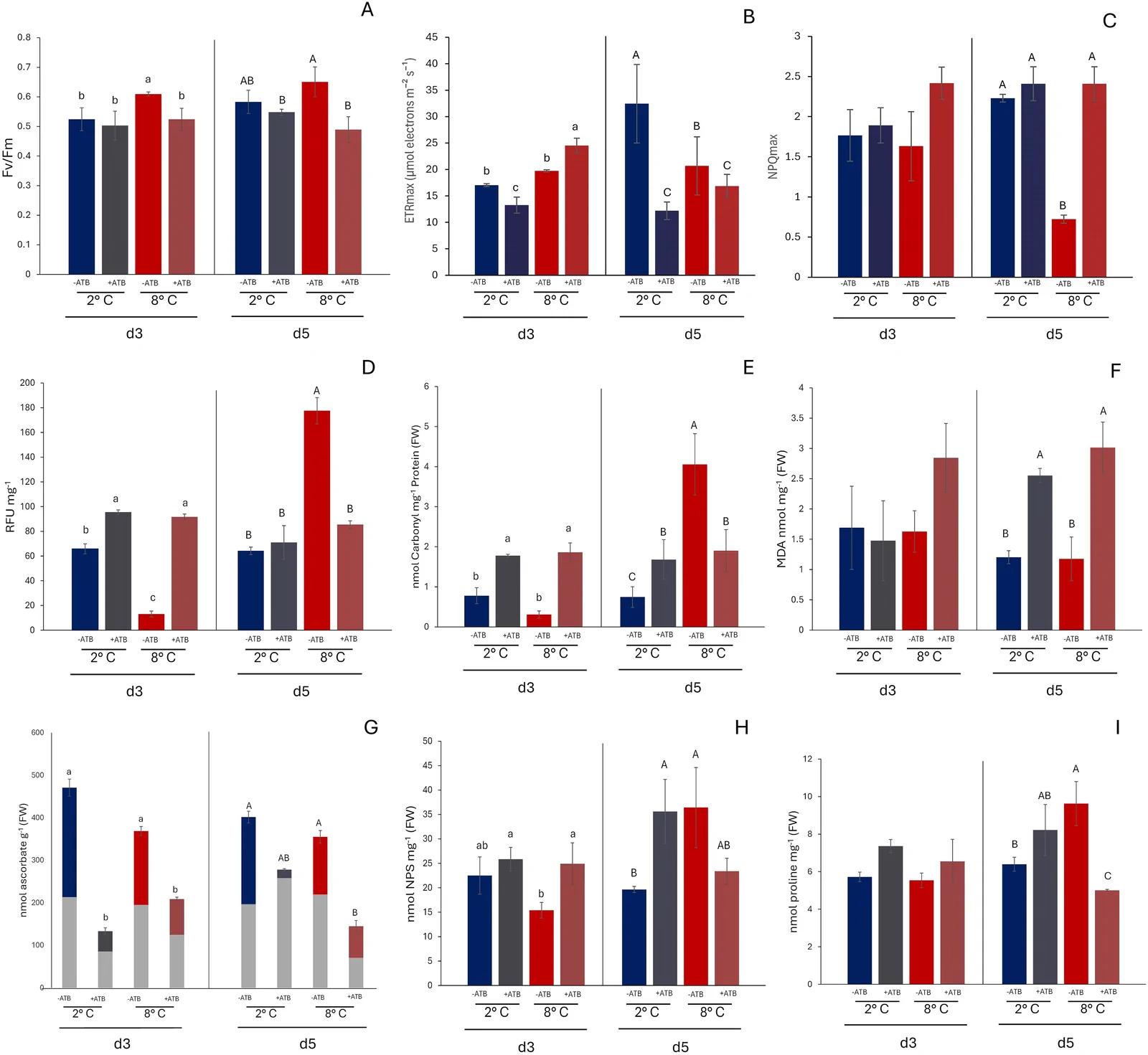
Health status in *A. utricularis* under thermal exposure and microbiome disruption. Photosynthetic parameters and cellular stress biomarkers were assessed in *A. utricularis* under an attenuated/disrupted microbiome using an antibiotic cocktail (+ATB). (A) Maximum quantum yield (F_v_/*F_m_*). (B) Maximum ETR (ETR_max_). (C) Maximum NPQ (NPQ_max_). (D) Total ROS. (E) Protein carbonylation. (F) Lipid peroxidation. (G) Total ascorbate showing in grey the oxidized (grey) form of ascorbate. (H) Free amino acids. (I) Proline. All graphs are presented as mean ± SD (*n* = 3, *P* < .05). Different letters indicate statistically significant differences between samples (HSD test, *P* < .05).

Regarding *A. utricularis* health status, photosynthetic performance *F_v_/F_m_* decreased significantly at 8°C + ATB in both days (Fig. 4A). No significant effect on ETR_max_ was observed after 3 days; nevertheless, by Day 5, elevated temperature caused a marked decline in ETR_max_, which was further exacerbated by the +ATB under both temperature conditions (Fig. 4B). NPQ_max_ decreased significantly at 8°C, while +ATB caused significant increase only by Day 5 (Fig. 4C). Host cellular stress under 8°C + ATB was evaluated by measuring oxidative stress and damage (Fig. 4D–I). The quantification of total ROS showed a significant decrease at 8°C after 3 days, although +ATB significantly increased ROS levels at both temperatures (Fig. 4D). Conversely, after 5 days, ROS levels significantly rise at 8°C, whereas +ATB showed maintained low ROS levels (Fig. 4D). Similarly, carbonylated proteins showed similar pattern to ROS levels (Fig. 4E). On the other hand, lipid peroxidation showed no significant differences after 3 days, although an increasing trend in membrane oxidative damage was observed exclusively in samples at 8°C + ATB (Fig. 4F). After 5 days, +ATB samples exhibited a significant increase in lipid damage at both temperatures. Antioxidant defenses revealed that ascorbate accumulation did not show significant differences between temperatures on either day (Fig. 4G). However, +ATB significantly decreased ascorbate accumulation at both temperatures. After 5 days, this reduction was only significant in samples at 8°C + ATB (Fig. 4G). Reduced/oxidized ascorbate ratio remained similar in all cases. Free amino acids levels showed that after 3 days, 8°C leads to a significant decrease, while +ATB does not induce changes compared to 2°C (Fig. 4H). However, after 5 days, +ATB showed different responses, while at 2°C there was a significant increase, at 8°C, a decreasing trend was observed. Regarding proline content, no significant differences were observed between treatments after 3 days (Fig. 4I). However, after 5 days, proline accumulation was higher at 8°C, although +ATB induced an increment in proline levels at 2°C, at 8°C, a drastic reduction was observed (Fig. 4I).

### *Adenocystis utricularis* epiphytic metabolome characterization under thermal stress

**Figure 5:**
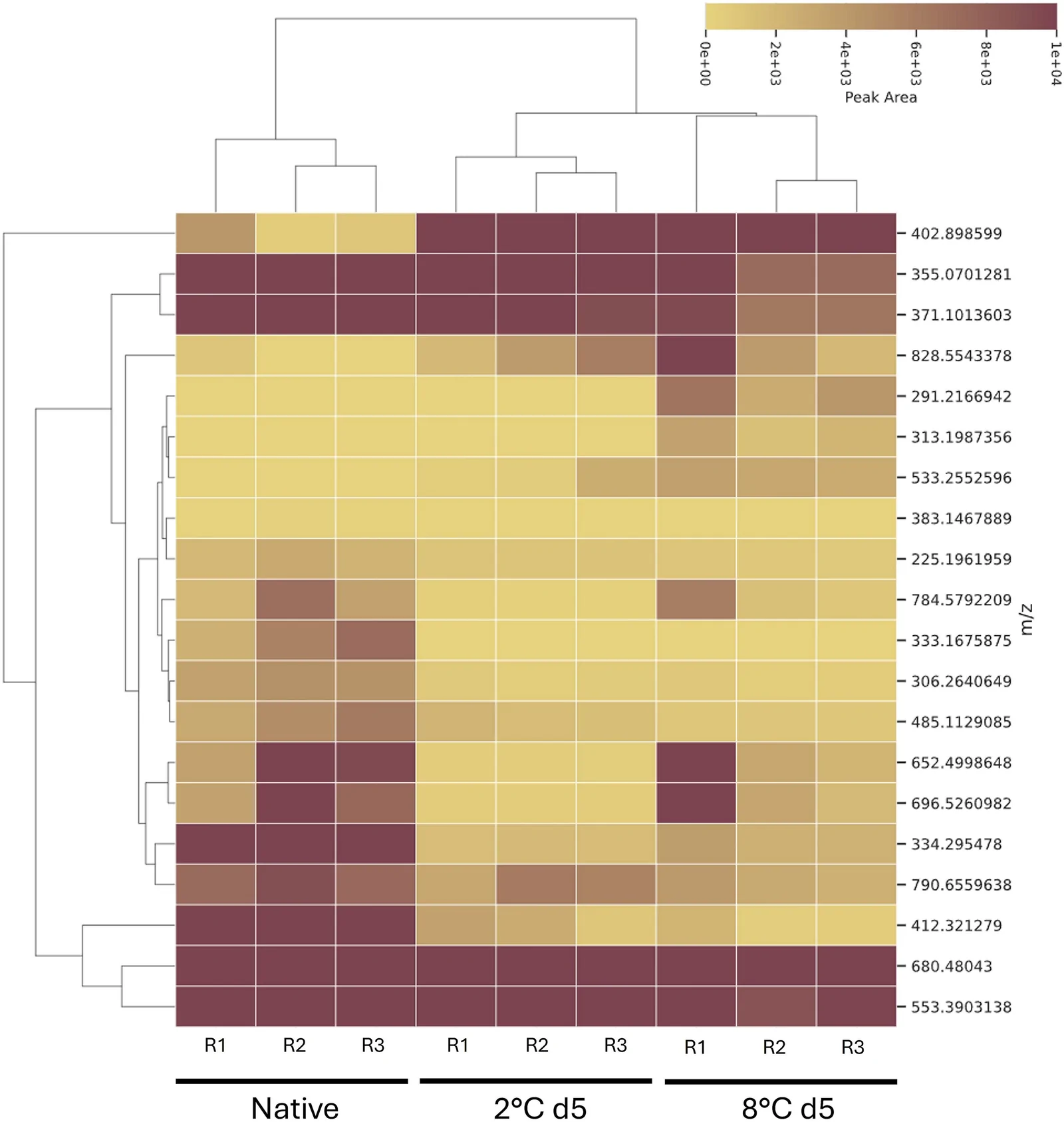
Comparison of the surface metabolome of *A. utricularis* throughout different conditions. Heatmap of the top 20 features with higher intensities (peak area values) detected by high-resolution tandem mass spectrometry in Native; experimental temperature = 2°C; and experimental temperature = 8°C.

Metabolic profiling of the experimental conditions of temperature (2°C and 8°C, at d5) were explored and compared within each other and in respect to the native control (samples collected *in situ* directly from the intertidal meadow), with a temperature of 2°C. PCA analysis showed a higher variability of metabolites in the native control, while differences between the two experimental conditions were less pronounced (Supplementary Fig. 4A). Hierarchical clustering of the 20 features with higher intensities confirmed further metabolic distinctive profiles among treatments (Fig. 5). Metabolites displayed contrasting condition-specific responses were further subjected to dereplication via SIRIUS using CSI:FingerID + CANOPUS to gain knowledge of the chemical class. MS/MS fragmentation was insufficient to confirm structural identities, as the annotations obtained with SIRIUS were consistent with MSI level 4 confidence assignments (Table 1). Nevertheless, they represent features strongly associated with the thermal stress treatment: (i) *m/z* 333.1676 was abundant in native samples but undetected under experimental conditions; (ii) *m/z* 291.2167 and 313.1987, were markedly elevated at 8°C; and (iii) *m/z* 1278.3531, was the only metabolite that decreased significantly in response to higher temperature (Supplementary Fig. 4B, Fig. 5).

**Table 1:** Chemical dereplication of unknown metabolites that present a differential metabolic profiling.

*m/z*	RT^a^	Intensity^b^	Condition^c^	Molecular formulae	Score^d^ (%)	Chemical class^e^	Putative STRUCTURE	MSI
291.217	0.96	4.22e3	↑ 8°C	C_15_H_30_O_5_	93.3	*n/i*	-	4
296.295	3.29	9.39e6	↑ 2°C & 8°C	C_19_H_37_NO	37.5	N-acyl amine	Octadecylisocyanate (CSI:FingerID)	3
313.199	0.96	2.28e3	↑ 8°C	C_15_H_30_O_5_	74.7	*n/i*	-	4
324.326	4.06	6.67 e5	↑ 8°C	C_21_H_41_NO	100	N-acyl amine	N-Octadecylacrilamide (CSI:FingerID)	3
333.168	1.04	5.07e3	↑ Native	C_17_H_26_O_5_	81.4	*n/i*	-	4
399.358	3.29	1.01e7	↑ 8°C	C_23_H_46_N_2_O_3_	90.3	N-acyl alpha amino acid	Pendecamine (CSI:FingerID)	3
402.899	5.22	5.47e4	↑ 2°C & 8°C	C_11_H_12_Cl_6_O_3_	33.7	halobenzenes	Bis(5,5,5-trichloropent-1-en-3-yl) carbonate (CSI:FingerID)	3
427.389	4.06	5.07e6	↑ 2°C & 8°C	C_25_H_50_N_2_O_3_	62.8	N-acyl alpha amino acid	N-(Carboxymethyl)-N,N-dimethyl-3-(octadecanoylamino)propan-1-aminium (CSI:FingerID)	3
478.322	3.8	6.92e6	↑ 2°C	C_33_H_39_N_3_	65.7	diphenylmethanes	4-[[4-(diethylamino)phenyl]-(4-ethyliminonaphthalen-1-ylidene)methyl]-N,N-diethylaniline (CSI:FingerID)	3
564.535	7.35	0.33e5	↑ 8°C	C_36_H_69_NO_3_	100	long-chain ceramides	(Z)-N-[(E)-1,3-dihydroxyicos-4-en-2-yl]hexadec-7-enamide (CSI:FingerID)	3
590.427	3.94	3.09e6	↑ 8°C	C_31_H_59_NO_9_	76.6	*n/i*	-	4
634.453	3.94	1.11e3	↑ 8°C	C_33_H_63_NO_10_	38.90	*n/i*	-	4
678.479	3.94	8.96e5	↑ 8°C	C_34_H_65_N_5_O_7_	50.2	*n/i*	-	4
716.552	4,23	3.15 e5	↑ 8°C	C_37_H_73_N_5_O_8_	99.7	peptides	tert-butyl N-[8-amino-2-[[(2-methylpropan-2-yl)oxycarbonyl-[1-[(2-methylpropan-2-yl)oxycarbonylamino]butyl]amino]methyl]octyl]-N-[1-[(2-methylpropan-2-yl)oxycarbonylamino]butyl]carbamate (CSI:FingerID)	3
1278.353	7.77	6.93e1	↓ 8°C	C_58_H_75_N_3_O_17_S_6_	98.6	*n/i*	-	4

a RT for retention time, calculated in minutes.

b Calculated as the average of three individual experimental replicates. When metabolite appears in two conditions, the higher value is presented.

c As the condition where the metabolite shows a differential metabolic profiling, as follows: ↑ high intensity, ↓ low intensity, compared to the rest of conditions.

d SIRIUS confidence score (%) associated with the proposed molecular formula.

e Based on SIRIUS dereplication, obtained when a proper MS/MS pattern was available in data, otherwise stated as *n/i*, which stands for not identified.

Regarding the known chemical space, MetaboAnalyst revealed seven metabolites with a Student’s *t-*test statistically significant metabolic profile in thermal stress scenario, highlighted in the VIP analysis (Supplementary Fig. 4C). Only four of them (*m/z* 324.3263; 427.3896; 564.5354; 716.5524) were identified with an MSI 4 confidence level, meaning that it was possible to assign structural motifs to a chemical class and obtain a putative structure of them. Their respective chemical classes correspond to N-acyl amines, N-acyl alpha, amino acids, long-chain ceramides, and peptides, respectively (Table 1). Conversely, the remaining ones (*m/z* 590.4267; 634.4529; 678.4790) only were assigned with an MSI 4 confidence level, meaning that only a tentative molecular formula was assigned (Table 1). More importantly, 86% of these metabolites showed an increase intensity associated to thermal stress condition (8°C).

**Figure 6:**
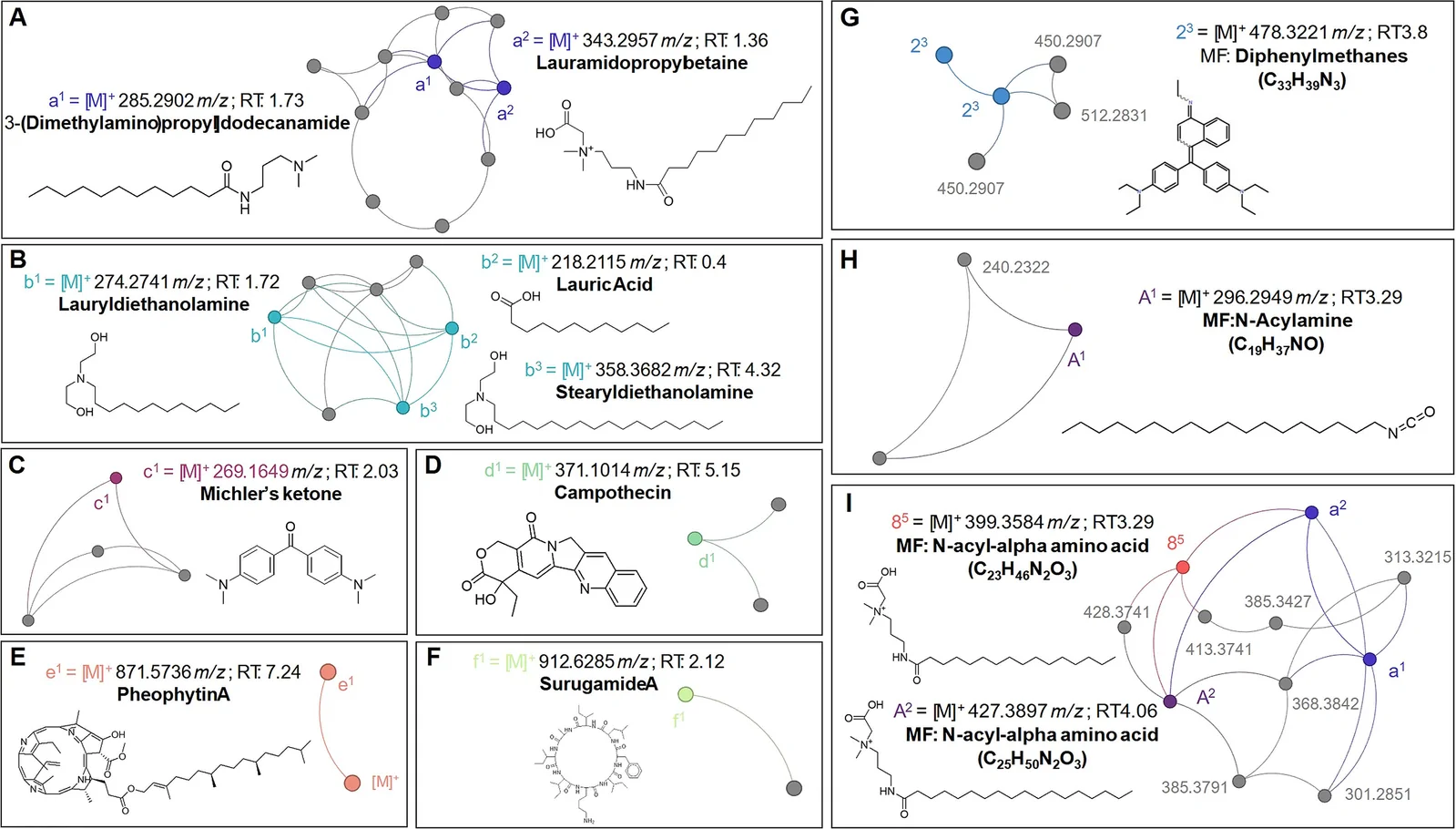
Surface metabolome dereplication of *A. utricularis.* GNPS-based molecular networking showing the dereplication of the known chemical space (panels A–F) and the unknown chemical space (panels G–I). Each panel represents a molecular network in which nodes correspond to MS/MS features, annotated with their *m*/*z* values, retention time (RT), and proposed chemical structures when available. Metabolite annotations were supported by GNPS library matches and complemented with SIRIUS predictions when possible. For the unknown chemical space, features were labeled according to their relative abundance across treatments: “2^3^” indicates metabolites enriched at 2°C , “8^5^” indicates metabolites enriched at 8°C , and “A^1^” and “A^2^” represent metabolites detected in both conditions. MF denotes molecular family.

On the other hand, within the unknown chemical space, features were subjected to GNPS library dereplication to explore further the vast chemical space. FBMN retrieved 267 nodes, where six subnetworks presented at least one node with a match in the database (colored nodes, Fig. 6). Interestingly, the largest networks corresponded to metabolites of synthetic origin (networks A–C, Fig. 6). For instance, network A and B are constituted by 3-(dimethylamino)propyl dodecanamide, lauramidopropyl betaine, lauryldiethanolamine, lauric acid, and stearyldiethanolamine, amphoteric molecules structurally resembling surfactants. Additionally, network C included a node matching Michler’s ketone, an anthropogenic compound commonly used in dyes and pigments. On the other hand, networks D–F are of natural origin associated to molecules with reported biological activity, such as camptothecin, pheophytin, and surugamide with cytotoxic, antioxidant, and antibiotic properties, respectively (Fig. 6). A heatmap of the metabolites annotated in the A–F networks revealed distinct abundance patterns in the temperature conditions (Supplementary Fig. 5). For example, surugamide (network F, node f^1^: *m/z* 912.6285) was not detected in native samples but more abundant at 2°C compared to 8°C. Similarly, lauryldiethanolamine (network B, node b^1^:*m/z* 274.2741) showed increased abundance at 2°C, whereas 3-(dimethylamino) propyl dodecanamide (network A, node a^1^:*m/z* 285.2902) was more prominent at 8°C (Fig. 6 and Supplementary Fig. 5).

The metabolomic landscape of *A. utricularis* remains mostly uncharacterized, as ~98% of nodes remained unannotated, comprising 208 features. Within them, 29 and 27 metabolites exhibited higher intensities at 8°C and 2°C, respectively (Supplementary Fig. 6). A scatter plot revealed four particularly distinctive features: (i) feature *m/z* 478.3221 most abundant at 2°C (network G, node 2^3^); (ii) feature *m/z* 399.3584 most abundant at 8°C (network I, node 8^5^); and (iii) features *m/z* 296.2949 (network H, node A^1^) and *m/z* 427.3897 (network I, node A^2^) both enriched under experimental conditions compared to the native control (Fig. 6 and Supplementary Fig. 6). An attempt to chemically characterize these four metabolites was performed with SIRIUS. Dereplication of high-resolution fragmentation patterns showed that feature 2^3^ belongs to the molecular family of the diphenylmethanes, feature A^1^ belongs to the N-acylamine chemical class; and both feature 8^5^ and A^2^ belong to the N-acyl-alpha amino acid family (Fig. 6).

### Chemical–taxonomic contextualization of surface metabolites

**Figure 7:**
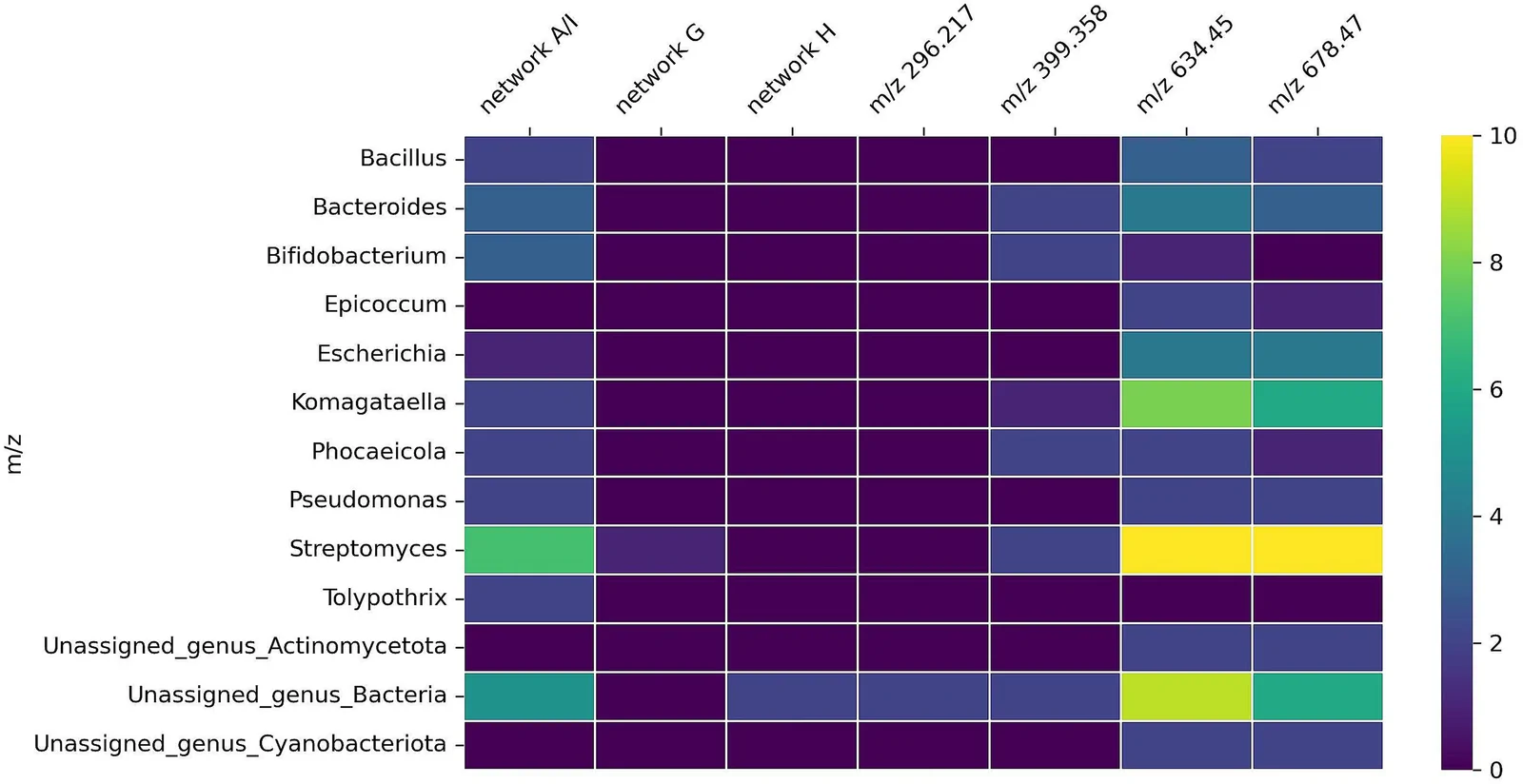
Chemical–taxonomic co-occurrence patterns of temperature-responsive surface metabolites in *A. utricularis*. Heatmap illustrating MicrobeMASST-based co-occurrence between molecular networks detected in the surface metabolome of *A. utricularis* and curated bacterial genera across public mass spectrometry datasets. Color intensity indicates the number of independent datasets in which each Network × Genus association was observed. Bacterial genera are presented to provide an integrated chemical–taxonomic context for temperature-responsive surface metabolites.

To contextualize the surface metabolomic landscape of *A. utricularis* with the microbiome patterns observed under thermal stress, we performed a MicrobeMASST-based taxonomic contextualization of selected MS/MS features. MS/MS spectra corresponding to selected surface-associated features were grouped into molecular networks based on FBMN and queried against public datasets to assess their taxonomic co-occurrence. The resulting Network × Genus heatmap summarizes the recurrence of these molecular networks across independent MassIVE datasets, with color intensity reflecting the number of datasets in which each network–taxon association was observed (Fig. 7). Distinct taxonomic associations were observed at the level of molecular families, being possible to assign metabolic features to 10 genera. Network A/I, G, and H composed by metabolites belonging to the N-acyl-alpha amino acid family that resemble surfactants, diphenylmethanes, and N-acyl amines families showed an enrichment in *Streptomyces* sp. and other unassigned bacteria genus (Fig. 7). On the other hand, metabolites *m/z* 296.217 and *m/z* 399.358, not belonging to networks but dereplicated as N-acyl amine and N-acyl alpha amino acids, respectively were present in *Bacteroides*, *Bifidobacterium*, *Komagataella*, *Phocaeicola*, and *Streptomyces* bacterial genus. Interestingly, the not-identified metabolites *m/z* 634.45 and 678.47 were particularly enriched in *Komagataella* and *Streptomyces* (Fig. 7).

## Discussion

To the best of our knowledge, this is the first study to characterize the epiphytic microbiome of an Antarctic macroalga and to evaluate the potential role of its microbial community in mediating host tolerance to acute thermal stress, including climate change-related warming scenarios and short-term extreme temperature events such as marine heatwaves. By integrating microbial community profiling, physiological stress assessments, and surface metabolomic analyses, our work provides a comprehensive and interdisciplinary framework for understanding holobiont resilience in polar environments.

### *Adenocystis utricularis* epiphytic microbiome structure dynamics under thermal stress

Alpha diversity exhibited a transient decline followed by partial recovery, which, together with temporal shifts in community composition and predicted functional profiles, is consistent with a short-term destabilization and reassembly of the epiphytic microbiome [22]. Such dynamics are consistent with microbial community responses to environmental disturbance, where diversity fluctuations and compositional restructuring occur before communities stabilize into a new configuration. While these patterns may resemble aspects of the “Anna Karenina Principle” (AKP), which predicts increased stochasticity in microbiomes under stress [16], our results primarily indicate compositional restructuring rather than a clear increase in community dispersion upon thermal stress condition. Similar transient responses have been reported in thermally stressed corals [42] and in the brown alga *Laminaria digitata*, where elevated seawater temperature altered the microbial composition and temporary shifts in diversity before community reassembly [43]. Multivariate analyses confirmed that experimental conditions (initial, 2°C and 8°C) significantly structured microbial communities. Across all treatments, core classes included Alphaproteobacteria, Bacteroidia, Gammaproteobacteria, and Verrucomicrobiae, consistent with prior studies in macroalgae holobionts [10, 12, 15, 44]. Exposure to 8°C was associated with an increased relative abundance of Campylobacteria and Gammaproteobacteria, while reducing Bacteroidia abundance, suggesting a shift toward taxa commonly associated with copiotrophic and fermentative metabolisms adapted to low oxygen environments [45, 46]. Within Gammaproteobacteria, genera such as *Colwellia*, *Psychromonas*, and *Paraglaciecola* possess versatile metabolic strategies, including fermentation, anaerobic via nitrate or sulphur respiration, and the synthesis of bioactive compounds [47–49]. These taxa are widely described as opportunistic marine bacteria whose ecological roles in macroalgal microbiomes are context dependent rather than inherently beneficial or pathogenic [50–52]. The class Campylobacteria (e.g. Arcobacteraceae genus) further suggests increased microbial processes linked to sulphur metabolism and organic matter degradation, potentially associated with algal tissue breakdown and DMSP turnover [53]. Conversely, the decline of Bacteroidia and potentially beneficial genera such as *Octadecabacter*, *Granulosicoccus*, *Algibacter*, *Arenicella*, and *Aurespira* suggests a transient loss of key ecological functions, including polysaccharide degradation [51, 54]. Discriminant ASV analysis highlighted heat-sensitive genera (*Arenicella*, *Aurespira*, and *Algibacter*) and the enrichment of resilient taxa such as *Colwellia* and *Psychromonas* which may thrive under stress conditions associated with elevated ROS levels at 8°C [55, 56]. Predicted functional profiles inferred from taxonomic assignments (FAPROTAX) mirrored these compositional shifts. Communities at 2°C were dominated by processes such as nitrogen fixation, sulphur oxidation, and cellulolysis, whereas those at 8°C showed higher potential for fermentation, nitrate reduction, photoheterotrophy, hydrocarbon degradation, and parasitic symbiosis, reflecting a stress-driven shift toward heterotrophic strategies [15, 57]. The increased relative abundance of Bdellovibrionota at 8°C further suggests that bacterial predators are natural members of these epiphytic communities and may respond rapidly to warming, as reported in other seaweed associated microbiomes [58]. Moreover, genomic evidence indicates that members of this group possess versatile adaptations allowing persistence in marine environments, including cold waters [59].

Taken together, our results suggest that warming intensified both bottom-up (substrate availability, host biomass) and top-down (predation, parasitism) processes, promoting a reorganization of microbial interactions on the algal surface. In this short-term context, functional restructuring of the epiphytic microbiome may contribute to maintaining holobiont performance under thermal stress, even though oxidative damage was not completely prevented. Importantly, the shift toward heterotrophy and bacterivory can be viewed as part of a broader reorganization of microbial interactions on the algal surface under thermal stress. Increased microbial turnover and recycling may enhance the processing of host-derived substrates and organic matter, potentially contributing to short-term functional buffering of the holobiont. At the same time, such reorganization could alter competitive and trophic balances within the epiphytic community, with possible consequences for taxa involved in antioxidant defense and nitrogen cycling. Overall, these observations underscore that early microbiome responses to warming involve dynamic reorganization rather than a uniform ecological shift.

### Photosynthetic performance and stress biomarkers in *A. utricularis* under thermal stress and microbiome disruption

Antimicrobial treatment as a perturbation tool to test holobiont microbial dependency significantly disrupted the activity of *Psychromonas*, *Colwellia*, and *Polaribacter*, and favored opportunistic taxa such as *Maribacter*, *Rubritalea*, *Octadecabacter*, *Lewinella*, and *Sulfitobacter*, known to thrive under thermal stress in disrupted microbiomes [60, 61]. Although subtle non-specific host effects of the antimicrobial cocktail cannot be entirely excluded, the markedly stronger physiological responses observed under combined warming and microbiome disruption suggest that stress amplification is primarily driven by microbiome restructuring rather than direct antibiotic toxicity. Indeed, broad-spectrum antibiotic treatments have been shown to transiently reduce microbial load and alter community structure in corals, sea anemones, and macroalgae, while maintaining host viability and physiological integrity [20–22]. In line with these observations, Vadillo González *et al*. [22] showed that perturbations in algal microbiomes under combined thermal and antimicrobial disruption promoted the proliferation of opportunistic taxa with broad metabolic potential, accompanied by a decline in functions related to nutrient cycling, vitamin synthesis, and oxidative stress regulation. Functional consequences of this microbial restructuring are particularly concerning, as predictive analyses indicated that the most affected pathways were related to nitrogen and sulphur cycling, as well as bacterivory. Functions such as nitrate reduction, denitrification, nitrogen respiration, and sulphur compound respiration are critical processes in polar coastal ecosystems, underpinning nutrient turnover and energy flow [62]. Their suppression under microbiome disruption suggests that the holobiont’s ability to mediate key biogeochemical processes may be significantly compromised.

Temperature emerged as the primary driver of photosynthetic performance in *A. utricularis*, exerting negative effects on *F_v_/F_m_*, ETR_max_, and NPQ_max_, consistent with previous findings [7]. Antimicrobial treatment effects became more evident after 5 days, suggesting synergistic stress responses in which microbiome disruption exacerbates thermal effects [63, 64]. As observed in corals, broad-spectrum antibiotics can disrupt beneficial microorganisms, leading to altered antioxidant defense and photoprotection [65, 66]. Microbial disruption has been linked to increased oxidative damage and altered photoprotective capacity, with specific taxa modulating *F_v_/F_m_*, ETR_max_ and NPQ responses [67]. Likewise, Paix *et al*. [68] demonstrated that synergistic effects of temperature and irradiance in the brown alga *Taonia atomaria* induced concurrent shifts in the epiphytic microbiota altering photosynthetic activity, oxidative stress regulation, and chemical defense. Microbial partners contribute to photosynthetic performance through ROS regulation, stress signaling, and antioxidant enhancement, roles compromised under antibiotic exposure [69]. Short-term thermal exposure (i.e. 3 days) did not induce significant oxidative stress and damage, in agreement with previous work on this species [7]; however, after 3d microbial disruption revealed evident oxidative stress in the host tissue, suggesting that microbiome integrity is fundamental for holobiont thermal stress tolerance [8]. After 5 days at 8°C microbial disruption did not increase ROS and protein carbonylation, suggesting a reconfigured microbiome under warming conditions, may reflect activation of repair and antioxidant production pathways, as previously observed elsewhere [70]. On the other hand, lipid peroxidation patterns were consistent with an association between native microbiota presence and reduced oxidative damage at 3 and 5 days. This observation aligns with studies on cold-adapted bacteria such as *Colwellia psychrerythraea*, whose metabolic flexibility, membrane adaptation, and polysaccharide production have been proposed to contribute to stress tolerance [55]. In our study, *Colwellia* abundance increased with temperature but decreased under antibiotics, supporting a potential functional relevance in the holobiont thermal response. Ascorbate levels, a key antioxidant, were unaffected by short-term warming but significantly reduced with antibiotic addition, particularly after 5 days at 8°C. This pattern suggests an association between microbiome disruption and reduced antioxidant capacity, consistent with coral studies reporting decreased antioxidant genes expression under antibiotic-induced dysbiosis [66] and observations that microbiome destabilization can precede host physiological decline [67]. Macroalgae also rely on osmolytes with antioxidant functions [71]. In *Laminaria solidungula*, mannitol depletion at high temperatures compromises osmotic and ROS-scavenging capacity [72]. Similarly, *A. utricularis* exposed thermal stress with a disrupted microbiome showed reduced osmolyte pools, including free amino acids and proline. Free amino acids declined after 3 days at elevated temperature, which may reflect increased metabolic demand rather than a direct effect of microbial loss, as suggested in corals [73]. Proline accumulation at 8°C supports its role in stress adaptation, yet this was impaired with antimicrobial addition. Together, these patterns indicate that microbiome disruption is associated with impaired osmolyte-related stress responses [74], although direct functional contributions of epiphytic bacteria require further experimental confirmation.

### Thermal stress impact on *A. utricularis* surface metabolome

Metabolomic profiling of *A. utricularis* revealed a predominance of uncharacterized chemical diversity, with more than 98% of detected MS/MS features remaining unannotated. While a degree of unknown chemical space is expected in untargeted metabolomics, the extent observed here likely reflects the limited representation of marine organisms in current spectral libraries, a gap that is even more pronounced for Antarctic macroalgae. This highlights not only a methodological limitation, but also the existence of a largely unexplored metabolomic landscape, suggesting that key compounds mediating ecological interactions at the algal surface remain undetected. Within this context, the fraction of annotated metabolites provides important insight into how the holobiont metabolome reorganizes under thermal stress. Using SIRIUS (integrating CSI:FingerID and CANOPUS), we identified 15 metabolites displaying distinct distribution patterns, with the majority (60%) enriched at 8°C. This shift supports the idea of an active metabolic response to elevated temperature, potentially linked to acclimation processes and holobiont resilience, in line with observations in thermotolerant corals [75, 76]. Network-based analyses further contextualized these patterns by revealing structured associations among metabolites. Several compounds were dereplicated as amphipathic molecules derived from fatty acids, including 3-dimethylaminopropyl dodecanamide, lauramidopropyl betaine, lauryldiethanolamine, lauric acid, and stearyldiethanolamine, all known for their surfactant properties [77]. These compounds may influence epibiotic community assembly by modulating surface physicochemistry and microbial attachment processes [78–81], thereby linking metabolomic shifts with potential changes in microbiome structure. Similar diethanolamine-derived surfactants have previously shown ecotoxicological effects in marine organisms, including algae, bivalves, and crustaceans [82], while recent HRMS-based environmental screenings have identified fatty acid-derived anthropogenic compounds, including lauric acid, among emerging substances of elevated toxicological and bioaccumulation concern in aquatic ecosystems [83]. Although evidence from Antarctic systems remains extremely limited, these findings suggest that such compounds may represent an underexplored component of emerging contaminant dynamics in polar coastal environments.

In addition, the detection of Michler’s ketone, an anthropogenic compound commonly used in dyes and pigments, points to the pervasive reach of pollutants even in remote Antarctic environments. This finding is consistent with previous reports of synthetic contaminants such as phthalates and siloxanes in polar ecosystems [84], reinforcing the need to consider external chemical pressures when interpreting metabolomic profiles in these systems. Such contaminants can reach Antarctic ecosystems through long-range atmospheric and oceanic transport, as well as localized inputs associated with research stations and tourism activities [85]. In this context, recent global-scale evidence shows that anthropogenic organic compounds are widely distributed across marine environments, from coastal to open ocean systems, and can represent a measurable fraction of the dissolved organic matter pool, even beyond direct sources [86]. Once deposited, these compounds may persist and accumulate in polar environments, where their physicochemical properties facilitate incorporation into food webs, potentially disrupting microbial interactions, host physiology, and broader ecosystem functioning through bioaccumulation and chronic exposure effects.

Beyond these compounds, GNPS dereplication also revealed bioactive metabolites of natural origin, including camptothecin, pheophytin, and surugamides. Camptothecin, a broad-spectrum antineoplastic alkaloid produced by endophytic bacteria such as *Bacillus*, *Lysinibacillus*, and *Paenibacillus* [87], along with pheophytin, known for its antioxidant properties in seaweeds [88], and surugamides, cytotoxic cyclic peptides derived from *Streptomyces* spp. [89, 90], suggest a functional contribution of the associated microbiota to the holobiont metabolome. These compounds may confer adaptive advantages related to stress mitigation, defense, or microbial competition, thereby contributing to holobiont stability under changing environmental conditions.

Overall, our findings support the view of the surface metabolome as a dynamic and responsive interface, where both host- and microbially derived compounds interact to mediate ecological processes. The coexistence of a vast unknown chemical space with targeted, stress-associated metabolic shifts underscores the complexity of holobiont responses to thermal perturbation and highlights the need for expanded metabolomic reference frameworks to better resolve these dynamics.

### Linking the chemical space to *A. utricularis* microbiome

The limited correspondence between taxonomic profiles and MS-based microbial contextualization underscores the inherent decoupling between microbial community structure and expressed chemical phenotypes within holobiont systems. While 16S rRNA meta-barcoding approaches capture community composition and genetic potential, subject to well-recognized amplification and protocol-dependent biases [91], untargeted metabolomics reflects an emergent chemical landscape shaped by host metabolism, microbial interactions, and temporal accumulation, within the constraints of incomplete reference libraries [92]. In this framework, MicrobeMASST provides a database-dependent approach to associate MS/MS features with potential microbial sources [41] and should therefore be interpreted as a hypothesis-generating tool rather than a direct assignment of in situ producers.

Consistent with this conceptual separation, the overlap between metabarcoding and metabolomic datasets was limited. Although 13 bacterial genera were identified as putative metabolite producers, only four (i.e. *Bacillus*, *Bacteroides*, *Bifidobacterium*, and *Pseudomonas*) were detected in our microbiome dataset. This restricted intersection does not necessarily indicate methodological limitations, but rather reflects the fact that taxonomic composition and chemical presence only partially converge. Similar patterns have been reported in seaweed holobionts, where shifts in surface metabolomes are not fully explained by community composition but instead reflect functional responses of associated microbiota [18, 68]. More broadly, the decoupling between microbial composition and functional output is a recurrent feature of microbial ecosystems (e.g. [31]), reinforcing the view that metabolite production is highly context-dependent and dynamically regulated.

Taken together, these findings highlight the importance of integrating multi-omics approaches to resolve the links between microbial identity and function, providing a more comprehensive understanding of how microbial communities shape the chemical landscape of the host.

## Conclusion

Our findings demonstrate the pivotal role of the epiphytic microbiome in shaping the stress response of *A. utricularis* under projected warming. Elevated temperature was associated with an early decline in microbial diversity, followed by a partial recovery of diversity metrics and an increased relative abundance of taxa commonly described as opportunistic, together with differences in predicted functional profiles. Warming favored metabolically versatile taxa (e.g. Campylobacteria, Gammaproteobacteria) while reducing taxa commonly associated with nutrient cycling, suggesting potential short-term functional compensation, but possible long-term instability. These microbial shifts were associated with changes in host physiology, with microbiome disruption leading to oxidative stress, depletion of antioxidant/osmotic defenses, and impaired photosynthesis, highlighting reduced capacity to maintain homeostasis. Surface metabolome analyses revealed largely uncharacterized metabolites, including microbial-derived bioactive compounds with possible protective functions, alongside unexpected detection of anthropogenic pollutants in this remote ecosystem. Together, this work provides the first integrated microbiome–metabolome characterization of an Antarctic alga, underscoring microbiome–host–chemical interactions as key mediators of these holobionts under climate change stressors.

## Supplementary Material

Supplementary_material_ycag208

## Data Availability

The datasets generated and/or analysed during the current study are available in the European Nucleotide Archive (ENA) repository (BioProject PRJEB109277) and the MetaboLights repository (accession MTBLS14103 https://www.ebi.ac.uk/metabolights/editor/MTBLS14103/files?reviewCode=937ea8b4-a4cf-4a49-9f8a-28a99e05577d).
