# Supplementary material for: Integrated holobiont responses of the Antarctic brown seaweed Adenocystis utricularis to thermal stress under a climate change scenario
Source: ISME Commun. 2026 Jul 21;6(1):ycag208. doi: 10.1093/ismeco/ycag208 (PMC13493149; doi:10.1093/ismeco/ycag208)
Supplement: Supplementary file 1 [file supplementary_material_ycag208.docx]

**Supplementary Material**

**2. Supplementary Methods**

**2.1 Experimental design**

**Supplementary Figure 1.** Experimental set up and microbiome sampling. **A**, Glass bottles with aerators placed in a temperature-controlled water bath. **B**, Close-up view of a glass bottle containing *Adenocystis utricularis*. **C,** Swabbing *A. utricularis* using a sterile cotton swab.

**2.2 Microbial community analyses**

Microbial community analyses were conducted in RStudio v4.2.2. Libraries were rarefied using the “rarefy_even_depth” function, and alpha diversity indices (species richness, Shannon index, and Pielou’s evenness index) were calculated with the “estimate_richness” and “evenness” functions from the “phyloseq” package [1]. The relative abundance of each sample was visualized through bar plots displaying the ten most abundant classes, while community composition changes at the genus level were analyzed using the “amp_heatmap” function from the “ampvis2” package [2].

To evaluate differences in microbial community structure across treatments, Bray-Curtis distances were computed, and their variation was analyzed through non-metric multidimensional scaling (NMDS) using the “ordinate” function from “phyloseq”. The assumption of homogeneous dispersions among groups was met, indicating that observed differences are attributable to treatment effects rather than dispersion.

Differential ASV analysis was performed with the “DESeq2” [3] package using non-normalized libraries to identify taxa with significant variations among treatments. Additionally, Taxonomy-based functional predictions associated with the ASVs present in the different samples were determined by using the “trans_func” function from the “*microeco”* package [4], specifying the “FAPROTAX” database for prokaryotic functional annotation [5].

**2.3 Photosynthetic analyses.**

*Photosynthetic activity assessed through in vivo Chlorophyll a fluorescence:* Photosystem II (PSII) activity was evaluated by measuring in vivo chlorophyll *a* fluorescence with a portable Junior PAM fluorometer and WinControl-3.2 software (Walz GmbH, Effeltrich, Germany). For each treatment, samples were incubated in 10 mL chambers, and three rapid light curves (RLCs) were obtained. RLCs provide information on PSII electron transport saturation and overall photosynthetic efficiency.

*Maximum Quantum Yield of PSII (Fv/Fm):* Rapid light curves (RLCs) were recorded after 15 min of dark adaptation by measuring basal fluorescence (Fo) and maximum fluorescence (Fm). Fo reflects the fluorescence emitted when all PSII reaction centers are oxidized, whereas Fm corresponds to the fluorescence signal when all centers are fully reduced.

*Electron Transport Rate (ETR):* The electron transport rate (ETR), as a proxy for photosynthetic performance and productivity, was determined after 20 s of exposure to twelve increasing levels of blue light irradiance (ranging from 25 to 1500 μmol photons m⁻² s⁻¹). ETR was calculated following [6] using the formula:

**ETR (μmol electron m⁻² s⁻¹) = ΔF/F'ₘ × E × A × FII**,

where ΔF/F'ₘ is the effective quantum yield (ΔF = F'ₘ − Ft; Ft is the steady-state fluorescence during actinic light, and F'ₘ is the maximum fluorescence after a saturating pulse), *E* is the incident PAR, *A* is thallus absorptance, and *FII* is the proportion of chlorophyll *a* allocated to PSII. Based on [7] and [8], *FII* was fixed at 0.8 for brown macroalgae [9]. Maximum ETR (ETRₘₐₓ) and the initial slope of the RLC (αETR), indicating the maximum photosynthetic capacity and light-use efficiency, respectively, were obtained by fitting data to the non-rectangular hyperbola model described by [10]. The light saturation point (EkETR) was calculated as ETRₘₐₓ divided by αETR.

*Non-photochemical quenching (NPQ):* NPQ used as a proxy for energy dissipation and photoprotective capacity, was calculated according to [11] as:

**NPQ = (Fm/Fm′) / Fm**

**2.4 Diagnostic biomarkers analyses**

*Total ROS quantification*: Following the protocol of [12], total ROS levels were fluorometrically quantified using the “Fluorimetric Intracellular ROS Kit” (Sigma-Aldrich) with 30 mg of ground algal biomass frozen in liquid nitrogen. Fluorescence measurements were carried out using a Cytation 5 reader (Agilent Biotek Instruments) with excitation at 540 nm and emission at 570 nm. Relative fluorescence units (RFU) were recorded under these settings.

*Lipid peroxidation quantification***:** Lipid peroxidation was assessed by quantifying thiobarbituric acid reactive substances (TBARS) as an indicator of oxidative damage, through malondialdehyde (MDA) concentration, following the protocol of [9]. Samples were analyzed in microplates at 532 nm using a spectrophotometer. The obtained concentrations were compared against a standard curve generated with commercial MDA (Sigma-Aldrich).

*Protein carbonyl quantification***:** Protein carbonyl groups were quantified following the method described by [13], with modifications for brown algae. Samples were measured in UV microplates and read at 366 nm in a spectrophotometer. Carbonyl concentrations were calculated using the Lambert-Beer equation with a molar extinction coefficient of 22,000 M⁻¹ cm⁻¹. Results were normalized to total protein content, which was quantified using the Bradford method [14].

*Free amino acid and proline quantification***:** Ninhydrin-positive substances (NPS) were quantified as an approximation of free amino acid and proline content to evaluate an additional stress response [12]. Samples were analyzed in microplates and read at 560 nm for free amino acids and 403 nm for proline using a spectrophotometer. The obtained concentrations were compared against a standard curve generated with a commercial free amino acid solution (Sigma-Aldrich).

*Ascorbate quantification***:** Ascorbate in its reduced (ASC) and oxidized (DHA) forms was quantified following the methodologies of [9] and [15], based on the reduction of the Fe (III)-TPTZ (2,4,6-tripyridyl-S-triazine) complex. ASC was measured at 593 nm in a spectrophotometer, the same wavelength used for total ascorbate (ASC + DHA) measurement. DHA content was obtained by subtracting reduced ascorbate from total ascorbate. The obtained concentrations were compared against a standard curve generated with commercial L-ascorbic acid (Sigma-Aldrich).

**2.5 Statistical analyses**

The normality of the data for biomarkers and photosynthetic parameters was assessed using the Shapiro-Wilk test, while homoscedasticity was evaluated with Bartlett’s test. When these assumptions were met, statistical analyses were conducted to identify significant differences among treatments. A two-way ANOVA was applied for physiological and biochemical variables, considering time and temperature as fixed factors, followed by Tukey’s HSD post hoc test when significant effects were detected. A significance level of α = 0.05 (p-value) was applied to all statistical tests. All tests were conducted in R 4.2.1 within RStudio 2024.12.0-467, with three biological replicates per treatment. The functions “aov” and “TukeyHSD” from the “stats” package were used for variance analysis and post hoc comparisons. For graphical representation of post hoc comparisons, significant differences among treatments were indicated using lowercase letters based on Tukey’s HSD test (p < 0.05). Treatments sharing at least one letter were not considered significantly different, whereas treatments with no letters in common differed significantly.

For microbiome-related univariate metrics (alpha diversity indices), statistical analyses were performed using one-way ANOVA, testing the effect of temperature separately at each sampling time. This approach was chosen to avoid overparameterization and to facilitate interpretation of treatment-specific effects. When assumptions of normality or homoscedasticity were not met, data were visually inspected and results interpreted with caution.

Differences in microbial community structure were assessed using permutational multivariate analysis of variance (PERMANOVA) based on Bray-Curtis dissimilarity matrices, implemented with the “adonis2” function from the “vegan” package. PERMANOVA tests were applied independently to evaluate the effects of temperature and antibiotic treatment on community composition. Homogeneity of multivariate dispersions was verified using the “betadisper” function prior to PERMANOVA. Ordination analyses (NMDS) were used exclusively for visualization purposes.

**2.6 Algae Surface Metabolome: Methodology Set Up**

Before extracting the Antarctic samples, a total of 9 g (wet weight) of frozen brown algae obtained from coastal Valparaíso **(Supplementary Figure 2A)** was used to set up an optimized extraction methodology. Surface metabolite extraction was slightly modified from a previous described protocol [16]**.** Briefly, frozen algae samples are directly immersed in an organic solvent of choice, a method known as solvent dipping. Dipping times of 5, 10, 15, 20, 25, 30 and 60 s were tested to identify the optimal duration that minimizes epidermal damage and limits the extraction of surface metabolites **(Supplementary Figure 2B)**. For each extraction, four frozen fronds of brown algae, previously adjusted by size and branching were immersed in 100 mL of a vigorously stirred n-hexane and methanol emulsion (1:1 v/v). Extracts were then evaporated under vacuum at 35 °C using a rotary evaporator, yielding approximately 2 mL of concentrated extract which was subsequently dried to constant weight in a pre-weighed vial. Each extraction per time was tested in triplicate. The samples were then analysed using a ultra-high performance liquid chromatography system (Dionex UltiMate 3000 UHPLC system, Thermo Fisher Scientific, USA), equipped with a diode array detector and a TSQ Quantis triple quadrupole mass spectrometer utilizing electrospray ionization in positive ion mode. Separation of the analytes was achieved using a Hypersil GOLD C18 column (100 × 2.1 mm, particle size 3 μm) from Thermo Fishers Scientific. The flow rate was set to 0.3 mL min⁻¹ and the column oven temperature was maintained at 35 °C. UV-detection was performed at a wavelength range from 210 to 600 nm. The ionization voltage was set to 3500 V, the ion transfer tube temperature to 350°C, and the vaporizer temperature to 400 °C. The mass spectrometry data acquisition was performed using scan mode at *m/z* 200-1500. Data processing, filtering and alignment (to conclude on the optimal time point for further extraction procedures) was conducted using MZmine v2.53 [17].

Comparison of the metabolite profiles obtained at the different extraction times **(Supplementary Figure 2C)**. identified 1 s as the optimal extraction time, maximizing the recovery of surface metabolites while minimizing the extraction of compounds released by algal tissue disruption. To our knowledge, this is the first protocol describing surface metabolome extraction from an Antarctic brown alga.

**Supplementary Figure 2. Algae surface metabolome methodology set up.** Establishment of an extraction protocol for *A. utricularis*. **A,** Trials were performed using a similar brown alga from Valparaíso Bay, considering similar amount of biomass. **B,** The extraction consisted of solvent-dipping approach, maintaining the algae in contact with the solvent mixture for several seconds. **C,** Extracts obtained from the different solvent-dipping times, after total evaporation before being subjected to UPLC for metabolic profiling characterization.

**2.7 *A. utricularis* surface metabolome extraction**

Extracts were analyzed using a Waters^TM^ Acquity UPLC BEH C18 (5 cm × 2.1 mm, 1.7-mm) column equipped with an Acquity UPLC BEH C18 guard column maintained at 70 °C. The mobile phases consisted of MilliQ^TM^ water + 10mM (v/v) ammonium acetate adjusted with NH_4_OH to reach pH=9 (A) and acetonitrile + 0.1% (v/v) formic acid (B) at a flow rate of 0.5 mL min^–1^. The chromatographic gradient applied was as follows: 40% B held for 0.4 min, followed by a linear gradient to 100% B over 5.6 min and held for 2.5 min before going back to initial conditions (the re-equilibration time was 1.5 min). The injection volume was set at 1 µL. The MS/MS measurements were done in positive-heated electrospray ionization (HESI) mode with a capillary voltage of 3500 V, acquiring in full MS/MS spectra mode (data dependent acquisition-driven MS/MS) with a *m/z* range of 100–1500. The MS1 resolution was set at 120,000 and the MS2 resolution at 30,000. Precursor ions were fragmented by stepped high-energy collision dissociation (HCD) using normalized collision energies of 20eV, 40eV, and 55eV. The automatic gain control (AGC) target value was set at 4×10^5^ for the full MS and 5×10^4^ for the MS/MS spectral acquisition.

**3. Results**

**3.1 Statistical results tables**

Results of univariate (ANOVA) and multivariate (PERMANOVA) statistical analysis performed on alpha and beta diversity metrics are provided in Supplementary Tables S1-S6. Alpha diversity was analyzed using one-way ANOVA testing temperature effects separately at each sampling time. Microbial community composition was assessed using PERMANOVA based on Bray-Curtis dissimilarities, and homogeneity of multivariate dispersion was evaluated using betadisper.

**Supplementary Table 1.** Results of one-way ANOVA assessing the effect of temperature on alpha diversity indices after 3 days of temperature exposure without ATB.

| Response variable | Factor | Df | Sum Sq | Mean Sq | F value | p-value |
| --- | --- | --- | --- | --- | --- | --- |
| richness | Temperature | 1 | 1291 | 1291 | 1.084 | 0.357 |
|  | Residuals | 4 | 4761 | 1190 |  |  |
| Shannon diversity | Temperature | 1 | 0.7670 | 0.7670 | 16.69 | 0.015 |
|  | Residuals | 4 | 0.1839 | 0.0460 |  |  |
| Pielou’s evenness | Temperature | 1 | 0.01895 | 0.01895 | 14.04 | 0.020 |
|  | Residuals | 4 | 0.00540 | 0.00135 |  |  |

One-way ANOVA results testing the effect of temperature (2°C vs 8°C) on alpha diversity metrics of the epiphytic microbiome after 3 days of exposure (n = 6) without ATB. Temperature had a significant effect on Shannon diversity and Pielou’s evenness, whereas richness did not differ significantly between treatments.

**Supplementary Table 2.** Results of one-way ANOVA assessing the effect of treatment (temperature × antibiotic condition) on alpha diversity metrics after 3 days of temperature exposure.

| Response variable | Factor | Df | Sum Sq | Mean Sq | F value | p-value |
| --- | --- | --- | --- | --- | --- | --- |
| richness | Temperature | 1 | 4648 | 4648 | 2.611 | 0.181 |
|  | Residuals | 4 | 7121 | 1780 |  |  |
| Shannon diversity | Temperature | 1 | 0.00042 | 0.00042 | 0.008 | 0.935 |
|  | Residuals | 4 | 0.22518 | 0.05630 |  |  |
| Pielou’s evenness | Temperature | 1 | 0.000806 | 0.000806 | 1.118 | 0.350 |
|  | Residuals | 4 | 0.002882 | 0.000721 |  |  |

One-way ANOVA results testing the effect of temperature on alpha diversity metrics of the epiphytic microbiome after 5 days of exposure (n = 11) without ATB showed no significant effect of temperature on richness, Shannon diversity, or Pielou’s evenness.

**Supplementary Table 3.** Effect of treatment (temperature × antibiotic condition) on alpha diversity metrics (n = 11 total samples).

| Response variable | Factor | Df | Sum Sq | Mean Sq | F value | p-value |
| --- | --- | --- | --- | --- | --- | --- |
| richness | Temperature | 3 | 8691 | 2897.1 | 3.596 | 0.074 |
|  | Residuals | 7 | 5639 | 805.6 |  |  |
| Shannon diversity | Temperature | 3 | 1.7452 | 0.5817 | 9.29 | 0.0078 |
|  | Residuals | 7 | 0.4384 | 0.0626 |  |  |
| Pielou’s evenness | Temperature | 3 | 0.04264 | 0.01421 | 7.655 | 0.013 |
|  | Residuals | 7 | 0.01300 | 0.00186 |  |  |

One-way ANOVA results testing the effect of treatment (temperature × antibiotic condition) on alpha diversity metrics of the epiphytic microbiome after 3 days of exposure (n = 11) showed a significant effect on Shannon diversity and Pielou’s evenness, whereas richness did not differ significantly among treatments.

**Supplementary Table 4.** Results of one-way ANOVA assessing the effect of treatment (temperature × antibiotic condition) on alpha diversity metrics after 5 days of temperature exposure.

| Response variable | Factor | Df | Sum Sq | Mean Sq | F value | p-value |
| --- | --- | --- | --- | --- | --- | --- |
| richness | Temperature | 3 | 17302 | 5767 | 5.421 | 0.0249 |
|  | Residuals | 8 | 8511 | 1064 |  |  |
| Shannon diversity | Temperature | 3 | 1.0077 | 0.3359 | 2.917 | 0.10 |
|  | Residuals | 8 | 0.9212 | 0.1151 |  |  |
| Pielou’s evenness | Temperature | 3 | 0.01913 | 0.00638 | 2.217 | 0.164 |
|  | Residuals | 8 | 0.02301 | 0.00288 |  |  |

One-way ANOVA results testing the effect of treatment (temperature × antibiotic condition) on alpha diversity metrics of the epiphytic microbiome after 5 days of exposure (n = 12) showed a significant effect on richness, whereas Shannon diversity and Pielou’s evenness did not differ significantly among treatments.

**Supplementary Table 5.** Results of PERMANOVA evaluating the effect of antibiotic treatment on microbial community composition.

| Factor | Df | Sum of Squares | R^2^ | F value | p-value |
| --- | --- | --- | --- | --- | --- |
| Antibiotic | 1 | 1.4521 | 0.3621 | 11.92 | < 0.001 |
| Residuals | 21 | 2.5583 | 0.6379 |  |  |
| Total | 22 | 4.0104 | 1.0000 |  |  |

PERMANOVA results based on Bray–Curtis dissimilarities evaluating the effect of antibiotic treatment on microbial community composition. Antibiotic treatment explained 36.2% of the variation in community structure and significantly altered the composition of the epiphytic microbiome.

**Supplementary Table 6.** Test results for homogeneity of multivariate dispersion (betadisper) assessing the effect of antibiotic treatment.

| Factor | Df | Sum Sq | Mean Sq | F value | p-value |
| --- | --- | --- | --- | --- | --- |
| Antibiotic | 1 | 0.0068 | 0.0068 | 1.1546 | 0.2948 |
| Residuals | 21 | 0.1238 | 0.0058 |  |  |
|  |  |  |  |  |  |

Analysis of multivariate homogeneity of dispersions (betadisper) based on Bray–Curtis dissimilarities. No significant differences in dispersion were detected between antibiotic treatments, indicating that PERMANOVA results reflect differences in community composition rather than differences in within-group variability.

**Supplementary Table 7.** PERMANOVA testing the effect of temperature (2 °C vs 8 °C) on microbial community +ATB composition based on Bray–Curtis dissimilarities (9999 permutations).

| Factor | Df | Sum OF Squares | R^2^ | F value | p-value |
| --- | --- | --- | --- | --- | --- |
| Temperature | 1 | 0.3578 | 0.0892 | 2.0568 | 0.0646 |
| Residuals | 21 | 3.6527 | 0.9108 |  |  |
| Total | 22 | 4.0104 | 1.0000 |  |  |

PERMANOVA results based on Bray–Curtis dissimilarities testing the effect of temperature on microbial community composition. Temperature explained 8.9% of the variation in community structure, although this effect was not statistically significant. However, significant differences in dispersion were detected between temperature treatments.

**Supplementary Table 8.** Test for homogeneity of multivariate dispersion (betadisper) assessing the effect of temperature on microbial community +ATB composition.

| Factor | Df | Sum Sq | Mean Sq | F value | p-value |
| --- | --- | --- | --- | --- | --- |
| Temperature | 1 | 0.0254 | 0.02539 | 8.4201 | 0.0085 |
| Residuals | 21 | 0.0633 | 0.00302 |  |  |

Analysis of multivariate homogeneity of dispersions (betadisper) testing whether variability in microbial community composition differed between temperature treatments.

**Supplementary Table 9.** PERMANOVA testing the effect of experimental condition (initial, 2°C and 8°C) on microbial community composition in the absence of antibiotic treatment.

| Factor | Df | Sum OF Squares | R^2^ | F value | p-value |
| --- | --- | --- | --- | --- | --- |
| Experimental condition | 2 | 0.7802 | 0.3620 | 3.4049 | <0.001 |
| Residuals | 12 | 1.3749 | 0.6380 |  |  |
| Total | 14 | 2.1551 | 1.0000 |  |  |

PERMANOVA results based on Bray–Curtis dissimilarities evaluating the effect of experimental condition (initial, 2°C and 8°C) on microbial community composition in the absence of antibiotic treatment. Experimental condition explained 36.2% of the variation in community structure and significantly influenced microbial composition.

**Supplementary Table 10.** Test for homogeneity of multivariate dispersion (betadisper) assessing the effect of experimental condition (initial, 2°C and 8°C) on microbial community composition in the absence of antibiotic treatment.

| Factor | Df | Sum Sq | Mean Sq | F value | p-value |
| --- | --- | --- | --- | --- | --- |
| experimental condition | 2 | 0.0320 | 0.0160 | 1.4729 | 0.2679 |
| Residuals | 12 | 0.1305 | 0.0109 |  |  |

Analysis of multivariate homogeneity of dispersions (betadisper) based on Bray–Curtis dissimilarities. No significant differences in dispersion were detected among experimental conditions (initial, 2°C and 8°C), indicating that variability in community composition was comparable across groups.

**3.2 Antimicrobial cocktail promote epiphytic bacterial microbiome disruption in *A. utricularis***

Taxonomy-based functional predictions of ATB treated samples at both temperatures (**Supplementary Figure 3**) revealed distinct shifts in the potential metabolic functions of the epiphytic microbiome associated with *A. utricularis* in response to temperature in a disrupted microbiome. Mos affected functions under +ATB were nitrate reduction, nitrate/nitrite denitrification, nitrogen respiration, nitrous oxide denitrification and methanol oxidation.

**Supplementary Figure 3.** Taxonomy-based functional profiles of bacterial communities associated with *A. utricularis* under ATB treatments and thermal exposure. Red indicates enriched functions, while blue indicates non-enriched functions across the different thermal treatments. T0 corresponds to day 0 post-acclimation, T1 to samples collected after 3 days, and T2 to those collected after 5 days. ATB indicates antibiotic cocktail treatment.

**3.3 *A. utricularis* surface metabolome characterization under thermal stress**

**Supplementary Figure 4.** **The Surface Metabolome of *A. utricularis.*** Comparison of the surface metabolome throughout the different conditions using MetaboAnalyst. **A,** Principal Component Analysis (PCA) plot, showing the differential metabolite profile between conditions: Blank, technical control (red); Control, native condition (green); Temp_2C, experimental temperature of 2°C (blue); and Temp_8C, experimental temperature of 8°C (light blue). **B**, Bar plots of the selected four metabolites that showed a contrasting metabolic profiling pattern within the tested conditions. **C**, Variable Importance Projection (VIP) analysis showing the metabolites responsible of the differentiation between conditions, indicated by colours. Blank, technical control; Native, natural settings; 2°C, experimental temperature = 2°C; and 8°C, experimental temperature = 8°C.

**Supplementary Figure 5. Surface Metabolome Dereplication of *A. utricularis.*** GNPS dereplication of the known chemical space. Heatmap illustrating the intensities (peak area values) of the dereplicated metabolites (letters from **A** to **F**) in the different conditions: Native; experimental temperature of 2°C; and experimental temperature of 8°C.

**Supplementary Figure 6. Surface Metabolome Dereplication of *A. utricularis*: The Unknown Chemical Space.** GNPS dereplication of the unknown chemical space, complemented with SIRIUS. Scatter plot (above) shows the patterns of all features based in m/z and intensities (peak area values). Venn diagram (middle) shows the selected metabolites and the condition where they appear as most abundant. Heatmap (below) showcase the comparison of the intensities (peak area values) in the different conditions tested: Native; experimental temperature of 2°C (blue); and experimental temperature of 8°C (red).

**REFERENCES**

**1.**	McMurdie PJ, Holmes S. phyloseq: An R Package for Reproducible Interactive Analysis and Graphics of Microbiome Census Data. *PLoS One* 2013;8:e61217. https://doi.org/10.1371/journal.pone.0061217

2.	Andersen KS, Kirkegaard R, Karst SM, Albertsen M. ampvis2: an R package to analyze and visualize 16S rRNA amplicon data. 2018. https://doi.org/10.1101/299537

3.	Love MI, Huber W, Anders S. Moderated estimation of fold change and dispersion for RNA-seq data with DESeq2. *Genome Biol* 2014;15:550. https://doi.org/10.1186/s13059-014-0550-8

4.	Liu C, Cui Y, Li X, Yao M. *microeco* : an R package for data mining in microbial community ecology. *FEMS Microbiol Ecol* 2021;**97**. https://doi.org/10.1093/femsec/fiaa255

5.	Louca S, Parfrey LW, Doebeli M. Decoupling function and taxonomy in the global ocean microbiome. *Science (1979)* 2016;353:1272–1277. https://doi.org/10.1126/science.aaf4507

6.	Schreiber U, Bilger W, Neubauer C. Chlorophyll Fluorescence as a Nonintrusive Indicator for Rapid Assessment of *In Vivo* Photosynthesis. Ecophysiology of Photosynthesis. Berlin, Heidelberg: Springer Berlin Heidelberg, 1995, 49–70.

7.	Orzymski J, Johnsen G, Sakshaug E. The significance of Intracellular Self‐Shading on the Biooptical properties of Brown, Red, and Green Macroalgae. *J Phycol* 1997;33:408–414. https://doi.org/10.1111/j.0022-3646.1997.00408.x

8.	Figueroa FL, Celis-Plá PSM, Martínez B, Korbee N, Trilla A, Arenas F. Yield losses and electron transport rate as indicators of thermal stress in *Fucus serratus* (Ochrophyta). *Algal Res* 2019;41:101560. https://doi.org/10.1016/j.algal.2019.101560

9.	Celis-Plá PSM, Moenne F, Rodriguez-Rojas F, Pardo D, Lavergne C, Moenne A, et al. Antarctic intertidal macroalgae under predicted increased temperatures mediated by global climate change: Would they cope? *Science of The Total Environment* 2020;**740**:140379. https://doi.org/10.1016/j.scitotenv.2020.140379

10.	Eilers PHC, Peeters JCH. A model for the relationship between light intensity and the rate of photosynthesis in phytoplankton. *Ecol Modell* 1988;42:199–215. https://doi.org/10.1016/0304-3800(88)90057-9

11.	Celis-Plá PSM, Bouzon ZL, Hall-Spencer JM, Schmidt EC, Korbee N, Figueroa FL. Seasonal biochemical and photophysiological responses in the intertidal macroalga *Cystoseira tamariscifolia* (Ochrophyta). *Mar Environ Res* 2016;115:89–97. https://doi.org/10.1016/j.marenvres.2015.11.014

12.	Pérez-Hernández G, Morales D, Pereira-Rojas J, Díaz MJ, Blanco-Murillo F, Sola I, et al. The halotolerant white sea anemone *Anthothoe chilensis*, highly abundant in brine discharges zones, as a promising biomonitoring species for evaluating the impacts of desalination plants. *Desalination* 2024;**581**:117612. https://doi.org/10.1016/j.desal.2024.11761213.	Cubillos VM et al. Temporal changes in environmental conditions of a mid-latitude estuary (southern Chile) and its influences in the cellular response of the euryhaline anemone *Anthopleura hermaphroditica*. *Ecol Indic* 2018;88:169–180. https://doi.org/10.1016/j.ecolind.2018.01.015

14.	Bradford MM. A rapid and sensitive method for the quantitation of microgram quantities of protein utilizing the principle of protein-dye binding. *Anal Biochem* 1976;72:248–254. https://doi.org/10.1016/0003-2697(76)90527-3

15.	Rodríguez-Rojas F, López-Marras A, Celis-Plá PSM, Muñoz P, García-Bartolomei E, Valenzuela F, et al. Ecophysiological and cellular stress responses in the cosmopolitan brown macroalga Ectocarpus as biomonitoring tools for assessing desalination brine impacts. *Desalination* 2020;489:114527. https://doi.org/10.1016/j.desal.2020.114527

16.	Rickert E, Wahl M, Link H, Richter H, Pohnert G. Seasonal Variations in Surface Metabolite Composition of *Fucus vesiculosus* and Fucus serratus from the Baltic Sea. *PLoS One* 2016;11:e0168196. https://doi.org/10.1371/journal.pone.0168196

17.	Pluskal T, Castillo S, Villar-Briones A, Orešič M. MZmine 2: Modular framework for processing, visualizing, and analyzing mass spectrometry-based molecular profile data. *BMC Bioinformatics* 2010;**11**:395. https://doi.org/10.1186/1471-2105-11-395
